# Three new Diplozoidae mitogenomes expose unusual compositional biases within the Monogenea class: implications for phylogenetic studies

**DOI:** 10.1186/s12862-018-1249-3

**Published:** 2018-09-03

**Authors:** Dong Zhang, Hong Zou, Shan G. Wu, Ming Li, Ivan Jakovlić, Jin Zhang, Rong Chen, Wen X. Li, Gui T. Wang

**Affiliations:** 1 Key Laboratory of Aquaculture Disease Control, Ministry of Agriculture, and State Key Laboratory of Freshwater Ecology and Biotechnology, Institute of Hydrobiology, Chinese Academy of Sciences, Wuhan, 430072 People’s Republic of China; 2 University of Chinese Academy of Sciences, Beijing, People’s Republic of China; 3 Bio-Transduction Lab, Biolake, Wuhan, 430075 People’s Republic of China

**Keywords:** Phylogenomics, Mutational bias, Molecular marker, Long branch, Amino acid usage, Ancestral gene order reconstruction

## Abstract

**Background:**

As the topologies produced by previous molecular and morphological studies were contradictory and unstable (polytomy), evolutionary relationships within the Diplozoidae family and the Monogenea class (controversial relationships among the Discocotylinea, Microcotylinea and Gastrocotylinea suborders) remain unresolved. Complete mitogenomes carry a relatively large amount of information, sufficient to provide a much higher phylogenetic resolution than traditionally used morphological traits and/or single molecular markers. However, their implementation is hampered by the scarcity of available monogenean mitogenomes. Therefore, we sequenced and characterized mitogenomes belonging to three Diplozoidae family species, and conducted comparative genomic and phylogenomic analyses for the entire Monogenea class.

**Results:**

Taxonomic identification was inconclusive, so two of the species were identified merely to the genus level. The complete mitogenomes of *Sindiplozoon* sp. and *Eudiplozoon* sp. are 14,334 bp and 15,239 bp in size, respectively. *Paradiplozoon opsariichthydis* (15,385 bp) is incomplete: an approximately 2000 bp-long gap within a non-coding region could not be sequenced. Each genome contains the standard 36 genes (*atp8* is missing). G + T content and the degree of GC- and AT-skews of these three mitogenome (and their individual elements) were higher than in other monogeneans. *nad2*, *atp6* and *nad6* were the most variable PCGs, whereas *cox1*, *nad1* and *cytb* were the most conserved. Mitochondrial phylogenomics analysis, conducted using concatenated amino acid sequences of all PCGs, indicates that evolutionary relationships of the three genera are: (*Eudiplozoon*, (*Paradiplozoon*, *Sindiplozoon*)); and of the three suborders: (Discocotylinea, (Microcotylinea, Gastrocotylinea)). These intergeneric relationships were also supported by the skewness and principal component analyses.

**Conclusions:**

Our results show that *nad2*, *atp6* and *nad6* (fast-evolving) would be better candidates than *cox1* (slow-evolving) for species identification and population genetics studies in Diplozoidae. Nucleotide bias and codon and amino acid usage patterns of the three diplozoid mitogenomes are more similar to cestodes and trematodes than to other monogenean flatworms. This unusual mutational bias was reflected in disproportionately long branches in the phylogram. Our study offsets the scarcity of molecular data for the subclass Polyopisthocotylea to some extent, and might provide important new insights into the evolutionary history of the three genera and three suborders.

**Electronic supplementary material:**

The online version of this article (10.1186/s12862-018-1249-3) contains supplementary material, which is available to authorized users.

## Background

As an adaptation to a broad range of dramatically different environments, monogenean flatworms often exhibit unique and exciting (from scientific perspective) life history strategies [1]. Monogeneans belonging to the Diplozoidae family are ectoparasitic on the gills of (mainly) freshwater cyprinid and characid fishes [2]. Like all monogeneans, these parasites have a direct life-cycle (no intermediate host), but all species (approximately 60) [3] in this family are characterized by their unique life-history, where two larvae (diporpae) permanently fuse into a pair, morphing into an X-shaped single organism [4, 5]. Although this feature makes them very interesting from the broader perspective of evolutionary biology, they mostly received scientific attention for the damage they cause to their hosts: the pair of central hooks and four pairs of clamps which they use to attach themselves onto the gills of host [6] causes mechanical damage to the gill filaments, which often results in secondary infections (bacterial, mycotic) and anemia [7, 8].

Taxonomy and phylogeny of diplozoids are mainly based on morphology (central hooks, clamps and spermatozoid ultrastructure), host fishes, and single molecular marker (usually ITS2) data [3, 6, 9–14]. Nevertheless, there are several limitations to these methods. As discussed in our recent paper [15], morphological traits are liable to exhibit homoplasy in (parasitic) microscopic animals, causing taxonomic and phylogenetic artifacts. Furthermore, morphological characters of phylogenetic importance for diplozoids (central hooks, clamps) develop gradually in different developmental stages [11]. Host-specificity also played an important role in previous diplozoid classification studies [11]. Although monogeneans are considered to be highly host-specific parasites [16], species belonging to the Diplozoidae family exhibit unusual heterogeneity in terms of host specificity [9, 17]. As a result of this host plasticity, wrong taxonomic conclusions are often reached when arbitrarily describing a diplozoid species using solely the criterion of host species. Examples of such artefacts are six Chinese *Paradiplozoon* species and two *Inustiatus* species, all of which parasitise on different hosts, but exhibit negligible interspecific genetic differentiation, which indicates that they are most likely conspecifics [3, 9, 12, 18].

Regarding the molecular markers traditionally used to infer the phylogeny of Diplozoidae, studies mostly relied on nucleotide sequence of the second internal transcribed spacer of ribosomal DNA (ITS-2 rDNA) [9, 11–14, 17, 19]. However, many of these studies reported that different analytical methods and datasets produce incongruent results [9, 12, 13]. As proposed for other commonly-used single molecular markers, such as 18S rRNA [20], it is highly likely that these problems were caused by the limited resolution of phylogenetic signal carried by the short (around 700 bp) ITS-2 rDNA sequence. This suggests that molecular markers that carry the amount of information sufficient to provide a much higher resolving power would have to be employed by future studies [20, 21]. Mitogenomes appear as a good candidate, as they can provide a phylogenetic resolution superior to the traditionally used molecular markers, and thus have been widely used to infer the phylogenetic relationships of metazoan lineages [22–24]. Apart from contributing genetic data for phylogenetic studies, the availability of mitochondrial genomes is also a prerequisite for meta-analyses aimed at decoding the mechanisms of genomic evolution [22, 25].

Metazoan mitochondrial genomes possess numerous characteristics that make them suitable for phylogenetic studies: abundance of mitochondria in animal tissues, maternal inheritance, absence of introns, small size of genomes, and a rapidly growing number of complete mitogenomes deposited in public databases [26, 27]. Simultaneously, mitogenomes have become an increasingly popular tool in population genetics [28], phylogenetics [29, 30] and diagnostics [21, 31] of parasitic flatworms (Neodermata). However, their applicability is still somewhat curbed by the fact that many taxonomic categories remain poorly or not at all represented. Among the three major groups of neodermatans, mitogenomic sequences of the class Monogenea account for merely 16.8% of available mitogenomes [15]. Furthermore, within the underrepresented Monogenea, mitogenomes are available for only three species of the subclass Polyopisthocotylea (syn. Polyonchoinea, see [26]). All three mitogenomes belong to species classified into the order Mazocraeidea (Monogenea); representing two of its five suborders: Microcotylinea and Gastrocotylinea. Relationships among the three monogenean suborders, the aforementioned two and Discocotylinea, are controversial [32, 33]. To supplement the sparse available mitogenomic material for the subclass Polyopisthocotylea and attempt to investigate the previously described internal phylogenetic controversies, we have sequenced and characterized the mitogenomes of three species belonging to three different genera of the Diplozoidae family (Discocotylinea suborder, Mazocraeidea order). Following this, we conducted a phylogenetic analysis using the three new mitogenomes and all 18 monogenean mitogenomes available in the GenBank. The aim of the phylogenetic analysis was to: (1) explore the debated intergeneric relationships of the family Diplozoidae; and (2) explore the debated relationships of the three Mazocraeidea suborders: Discocotylinea, Microcotylinea and Gastrocotylinea.

## Results

### Genome organization and base composition

**Fig. 1:**
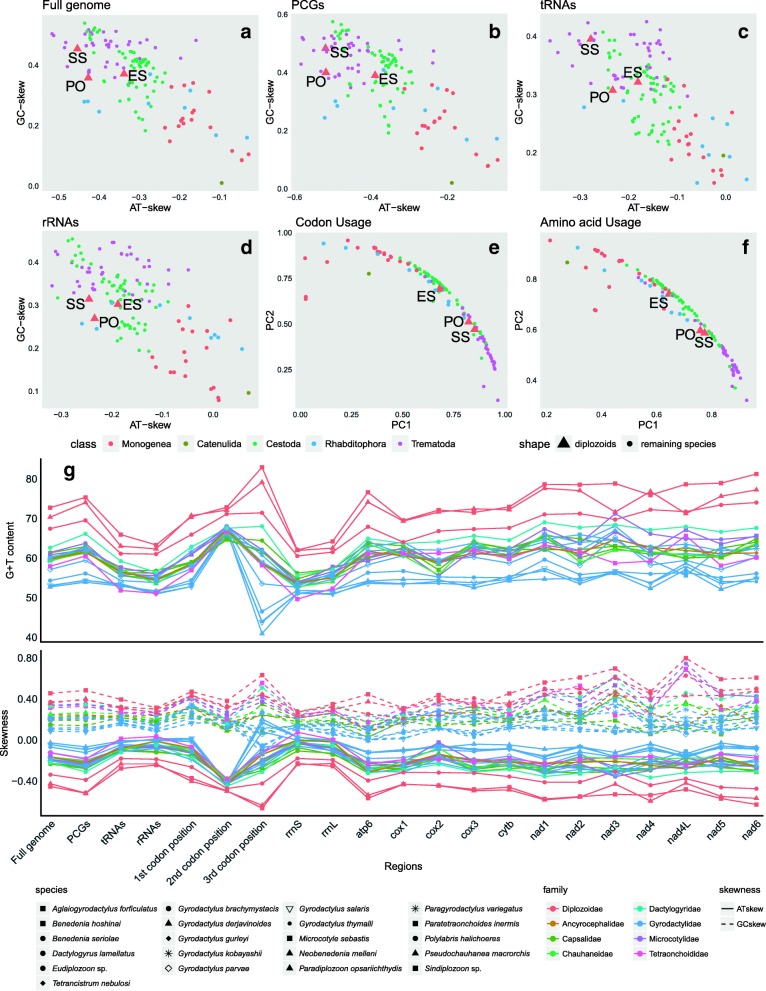
Nucleotide skews, codon usage, amino acid usage, and G + T content of the three studied mitogenomes, and comparison with other flatworms. **a-d** Comparison of nucleotide skews of the full genomes, protein-coding genes (PCGs), tRNAs and rRNAs for the mitogenomes of *Paradiplozoon opsariichthydis*, *Sindiplozoon* sp. and *Eudiplozoon* sp., and other flatworms. **e**, **f** Principal component (PC) analysis of the codon usage and amino acid usage in the PCGs of the three studied diplozoids and other flatworms. The first PC (PC1) and the second PC (PC2) of the codon usage and amino acid usage accounted for 95.8% and 94.7% of the variability, respectively. **g** G + T content and skewness of complete genomes and their individual elements. Species are colored according to their taxonomic placement at the class level in figure panels a-f and family level in the panel g. The three studied diplozoids are represented by triangles. ES: *Eudiplozoon* sp.; PO: *Paradiplozoon opsariichthydis*; SS: *Sindiplozoon* sp.

The full circular mitochondrial genomes of *Eudiplozoon* sp. and *Sindiplozoon* sp. are 14,334 bp and 15,254 bp in size (GenBank accession numbers are MG458328 and MG458326, respectively). The partial mitogenome of *Paradiplozoon opsariichthydis* Jiang, Wu & Wang, 1984 is 15,385 bp long (GenBank accession number MG458327). All of the typical 36 flatworm mitochondrial genes [23] were found in the three genomes, including twelve protein-encoding genes (PCGs), 22 tRNA genes, two rRNA genes (Additional file 1). All three mitogenomes lack the *atp8* gene, and all genes are transcribed from the same strand. The architecture, gene contents and similarity of orthologous sequences for the three studied mitogenomes are summarized in Table 1. Noteworthy, G + T content, as well as the degree of GC- and AT-skews, of the whole genome (and its individual elements) in all three studied species were higher than in other monogeneans (Fig. 1g).

**Table 1:** Comparison of the annotated mitochondrial genomes of *Paradiplozoon opsariichthydis*, *Sindiplozoon* sp. and *Eudiplozoon* sp.

Gene	Position		Size	Codon		Anti-codon	Identity
	From	To		Start	Stop		
*Eudiplozoon* sp. / *Paradiplozoon opsariichthydis* / *Sindiplozoon* sp.							ES-PO/ES-SS/PO-SS/A
*cox3*	1/1/1	756/780/762	756/780/762	ATG/ATG/ATG	TAA/TAG/TAA		60.13/56.49/53.91/56.85
*trnC*	755/763/821	819/822/888	65/60/68			GCA/GCA/GCA	56.92/76.47/63.24/65.54
*trnK*	826/826/897	894/890/961	69/65/65			CTT/CTT/CTT	55.71/53.52/69.70/59.64
*nad6*	1481/2320/2167	1931/2796/2643	451/477/477	GTG/ATG/GTG	T/TAA/TAG		52.41/57.29/52.85/54.18
*trnY*	1941/2776/2673	2003/2844/2738	63/69/66			GTA/GTA/GTA	59.42/71.21/68.12/66.25
*trnL1*	2004/2857/2766	2071/2923/2829	68/67/64			TAG/TAG/TAG	54.93/55.07/62.69/57.56
*trnS2*	2076/2926/2838	2136/2985/2896	61/60/59			TGA/TGA/TGA	65.57/68.85/66.67/67.03
*trnL2*	2137/2986/2900	2205/3052/2964	69/67/65			TAA/TAA/TAA	70.42/69.57/65.67/68.55
*trnR*	2207/3053/2966	2270/3116/3027	64/64/62			ACG/TCG/ACG	68.75/75.00/67.19/70.31
*nad5*	2271/3118/3002	3821/4657/4559	1551/1540/1558	TTG/ATG/GTG	TAG/T/T		54.54/61.90/55.63/57.35
*trnE*	3802/4658/4560	3868/4721/4626	67/64/67			TTC/TTC/TTC	39.47/69.57/52.24/53.76
*cytb*	3871/4722/4628	5016/5885/5803	1146/1164/1176	GTG/ATG/TTG	TAG/TAA/TAG		63.20/65.56/65.17/64.64
*nad4L*	5022/5876/5784	5279/6142/6059	258/267/276	GTG/ATG/GTG	TAG/TAG/TAG		62.17/55.43/55.67/57.76
*nad4*	5243/6152/6011	6484/7408/7261	1242/1257/1251	ATG/ATG/GTG	TAG/TAG/TAG		61.45/62.78/60.84/61.69
*trnF*	6493/7526/7390	6558/7591/7456	66/66/67			GAA/GAA/GAA	78.79/58.21/55.22/64.07
*trnQ*	6622/7606/7474	6683/7667/7535	62/62/62			TTG/TTG/TTG	58.06/66.13/59.68/61.29
*atp6*	6696/7676/7565	7277/8293/8140	582/618/576	GTG/ATT/ATG	TAA/TAA/TAG		49.84/55.56/53.55/52.98
*nad2*	7286/8339/8146	8161/9217/9033	876/879/888	ATG/ATT/TTG	TAG/TAG/TAG		53.96/55.64/56.67/55.42
*trnV*	8163/9218/9045	8226/9281/9111	64/64/67			TAC/TAC/TAC	50.00/64.18/60.87/58.35
*trnA*	8236/9282/9128	8300/9342/9191	65/61/64			TGC/TGC/TGC	56.92/72.31/64.06/64.43
*trnD*	8303/9344/9195	8368/9405/9263	66/62/69			GTC/GTC/GTC	60.61/52.11/62.32/58.35
*nad1*	8371/9407/9264	9294/10315/10185	924/909/922	ATG/ATG/TTG	TAG/TAG/T		63.69/68.17/65.30/65.72
*trnN*	9294/10320/10194	9358/10383/10256	65/64/63			GTT/GTT/GTT	61.19/68.18/62.50/63.96
*trnP*	9360/10388/10266	9423/10449/10327	64/62/62			TGG/TGG/TGG	78.12/76.92/75.00/76.68
*trnI*	9428/10451/10342	9495/10517/10410	68/67/69			GAT/GAT/GAT	82.35/73.91/62.32/72.86
*nad3*	9498/10521/10420	9794/10784/10698	297/264/279	ATG/ATG/GTT	TAA/TAA/TAG		56.25/64.00/56.89/59.05
*trnS1*	9802/10803/10701	9865/10870/10759	64/68/59			GCT/GCT/GCT	60.87/67.19/64.71/64.25
*trnW*	9867/10871/10760	9930/10935/10825	64/65/66			TCA/TCA/TCA	72.73/57.58/75.76/68.69
*cox1*	9940/10939/10827	11,511/12546/12399	1572/1608/1573	ATG/ATG/TTG	TAA/TAG/T		71.02/75.00/70.91/72.31
*trnG*	11,518/7417/12409	11,582/7479/12475	65/63/67			TCC/TCC/TCC	62.12/71.64/67.16/66.98
*trnT*	11,786/12873/12673	11,852/12933/12737	67/61/65			TGT/TGT/TGT	61.19/70.15/72.31/67.88
*rrnL*	11,853/12934/12740	12,826/13898/13718	974/965/979				73.98/78.34/73.96/75.43
*rrnS*	12,827/13899/13719	13,555/14633/14448	729/735/730				68.59/73.96/70.07/70.87
*cox2*	13,556/14634/14487	14,185/15260/15077	630/627/591	ATG/ATG/ATG	TAG/TAG/TAG		58.57/65.40/58.05/60.67
*trnM*	14,190/15259/15116	14,254/15322/15181	65/64/66			CAT/CAT/CAT	65.67/85.07/60.61/70.45
*trnH*	14,269/15323/15187	14,334/15383/15251	66/61/65			GTG/GTG/GTG	57.58/74.63/58.46/63.55

*ES Eudiplozoon* sp., *PO Paradiplozoon opsariichthydis*, *SS Sindiplozoon* sp., *A* average identity values (%) of the three diplozoids

### Protein-coding genes and codon usage

**Fig. 2:**
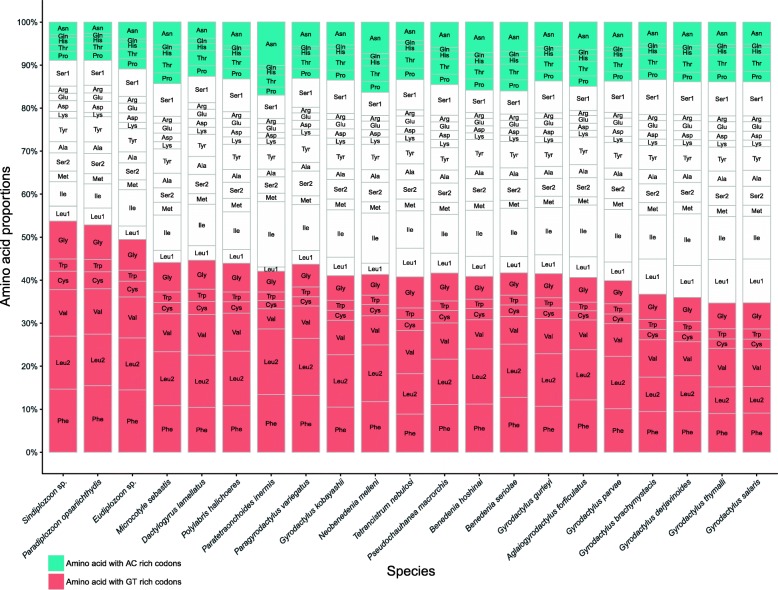
Amino acid composition of the 12 protein-coding genes in the mitogenomes of *Paradiplozoon opsariichthydis*, *Sindiplozoon* sp. and *Eudiplozoon* sp., and comparison to other monogenean species

Concatenated PCGs were 10,284, 10,389 and 10,326 bp in size, with A + T contents of 70.7%, 67.5% and 68%, for *Eudiplozoon* sp., *P. opsariichthydis* and *Sindiplozoon* sp. respectively. Third codon position exhibited the highest A + T bias (Additional file 2). Commonly used ATG and GTG initial codons were found in most of the PCGs of the three studied mitogenomes. However, it proved difficult to determine the start codons of several genes for the three species. As a working hypothesis, we proposed some presumptive initial codons based on the alignment with orthologs of other monogeneans: TTG for *nad5* in *Eudiplozoon* sp., ATT for *atp6* and *nad2* in *P. opsariichthydis*, and GTT (*nad3*) and TTG (*nad1*, *cytb*, *nad2* and *cox1*) in *Sindiplozoon* sp.. Most of the PCGs used the canonical TAA (includes the abbreviated T-- form) and TAG as stop codons (Table 1). Codon usage, RSCU, and codon family (corresponding to the amino acids) proportions were investigated among the six available polyopisthocotylid representatives (3 new + 3 old, Additional file 3). In comparison to other monogeneans, diplozoids exhibit a strong preference for amino acids encoded by guanine and thymine-rich codon families (such as Phe, Val and Gly), whereas amino acids encoded by adenosine and cytosine-rich codon families (such as Asn and Thr) appear to be selected against (Fig. 2 and Additional file 3). Principal component analyses (PCA) suggested that the overall codon usage of all three diplozoids and amino acid usage patterns of *P. opsariichthydis* and *Sindiplozoon* sp. were notably different from other monogeneans (Fig. 1e and f). Instead, they were more similar to trematodes and cestodes, in accordance with the results of nucleotide skewness (Fig. 1a-d). Within the Diplozoidae, the codon usage pattern of *Sindiplozoon* sp. was similar to that of *P. opsariichthydis* (Fig. 1e and f), which is also congruent with the results of nucleotide composition and skewness.

### Transfer RNA genes

**Fig. 3:**
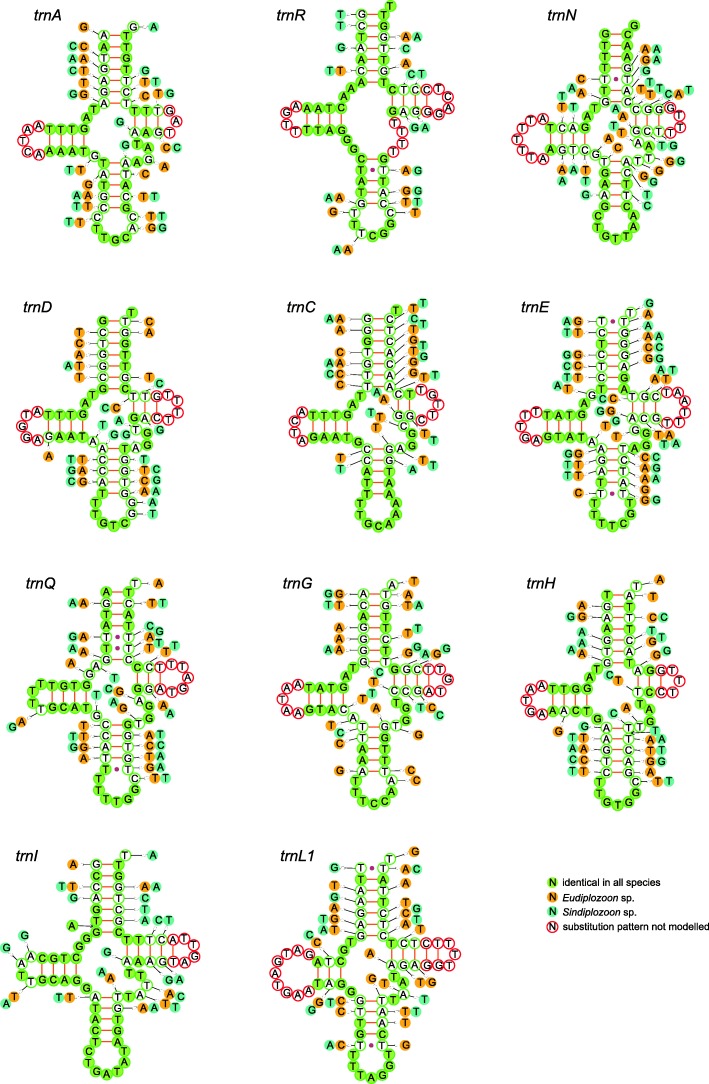
Secondary structures of tRNA families (*trnA*-*trnL1*) in diplozoid mitogenomes. Nucleotide substitution pattern for each tRNA family was modeled against the reference structure determined for *Paradiplozoon opsariichthydis*. Arrows in *trnH* and *trnI* denote a nucleotide insertion and an unmatched nucleotide in the acceptor stem

**Fig. 4:**
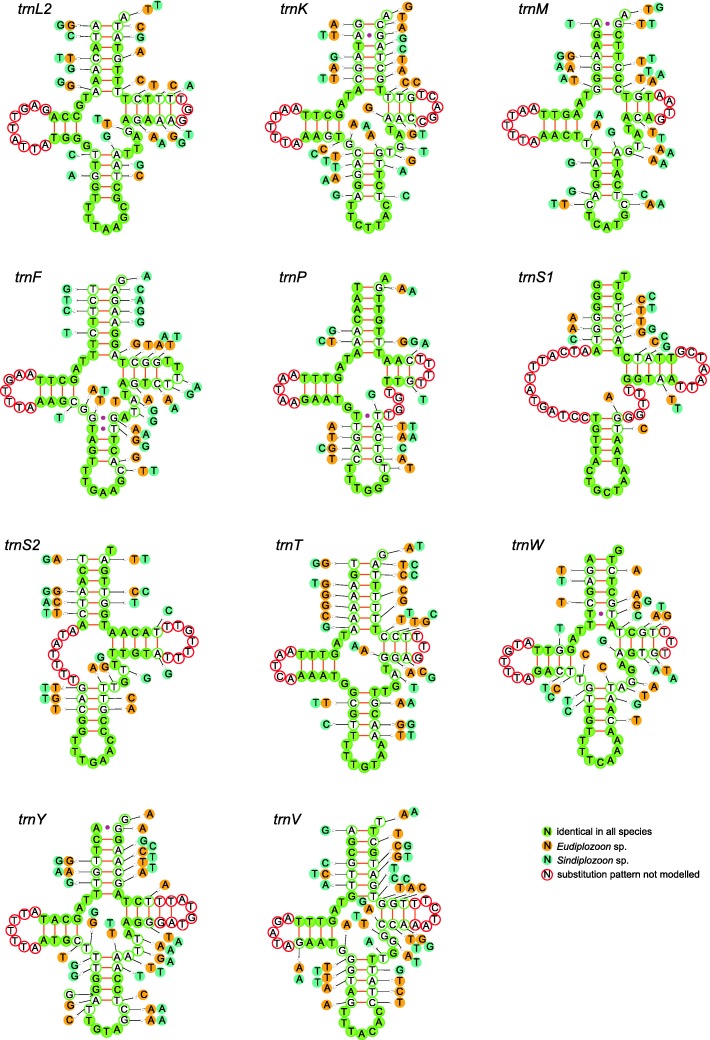
Secondary structures of tRNA families (*trnL2*-*trnV*) in diplozoid mitogenomes. Nucleotide substitution patterns were modeled against the reference structure determined for *Paradiplozoon opsariichthydis*

All 22 standard tRNAs were found (Table 1, Figs. 3 and 4), and most of them could be folded into the conventional cloverleaf structure (Figs. 3 and 4). Exceptions were *trnS1*^(AGN)^ and *trnS2*^*(UCN)*^, which lacked DHU arms (Figs. 3 and 4). The first site of anticodon sequences of all tRNAs was T or G, except for *trnM* and *trnK* in all three studied mitogenomes, and trnR in *Eudiplozoon* sp. and *Sindiplozoon* sp. (Figs. 3 and 4). The percentage of identical nucleotides among tRNA orthologs of the three mitogenomes (all three compared simultaneously) ranged from 40 (*trnK*) to 65 (*trnP*) (Additional file 4), which partially corresponds to the average identities of orthologous sequences (Table 1). Nucleotide substitutions observed among the tRNAs of the three diplozoid mitogenomes are mainly restricted to the TΨC and DHU loops, whereas the anticodon loop is highly conserved (Figs. 3 and 4). We also found a number of nucleotide substitutions in three different stems (acceptor, anticodon and TΨC), but most of them are compensatory base changes occurring in both paired nucleotides (cbc; i.e., G-C ↔ A-T) and/or hemi-cbcs (a mutation of a single nucleotide in a pair, while maintaining the nucleotide bond, e.g. G-T ↔ A-T) (Figs. 3 and 4). However, the DHU stem was extremely conserved, with only two cbcs, seven hemi-cbcs, one reverse change (i.e., A-T ↔ T-A) and one mismatched pair (T-T) among all 22 tRNAs (Figs. 3 and 4). Additionally, unmatched base insertions were observed in the stems of two tRNAs: there is an unmatched T base inserted in the anticodon stem of *trnH*, autapomorphic for *Sindiplozoon* sp., and an unmatched A base in the acceptor stem of *trnI*, which appears to be synapomorphic for diplozoids, and possibly even monogeneans (Fig. 3).

### Non-coding regions

All three studied mitogenomes, *Eudiplozoon* sp., *P. opsariichthydis* and *Sindiplozoon* sp., contain two large non-coding regions: a long non-coding region (LNR; 586, 1429 and 1205 bp respectively; note that the LNR of *P. opsariichthydis* is incomplete) and a short non-coding region (SNR; 203, 326 and 197 bp respectively). LNR and SNR of the three species are located between *trnK* and *nad6*, and *trnG* and *trnT*, respectively. Beyond this, there is an extra SNR (SNR1) in the mitogenome of *Sindiplozoon* sp.; 128 bp in size and located between *trnF* and *nad4L*. Tandem repeats (TRs) could not be found in any of the SNRs (including the SNR1 of *Sindiplozoon* sp.), but putative secondary structures containing a stem-loop were found in all of these sequences (Additional file 5). Several TR elements were predicted in LNRs of the three studied mitogenomes. A short TR region, composed of seven TRs, was found in the LNR of *Sindiplozoon* sp. Lengths and nucleotide composition of these seven repeat units were not highly conserved, but all were rich in A + T (Additional file 5). Within the LNR of *P. opsariichthydis* there was a large TR region (Additional file 5). Additionally, we found microsatellite-like elements in all three LNRs: TA_22_ in *Eudiplozoon* sp., TA_64_ (with four nucleotide mutations, one G insertion and one T/A position exchange) in *P. opsariichthydis*, and TA_20_ (with two mutations) in *Sindiplozoon* sp..

### Sliding window analysis and nucleotide diversity

**Fig. 5:**
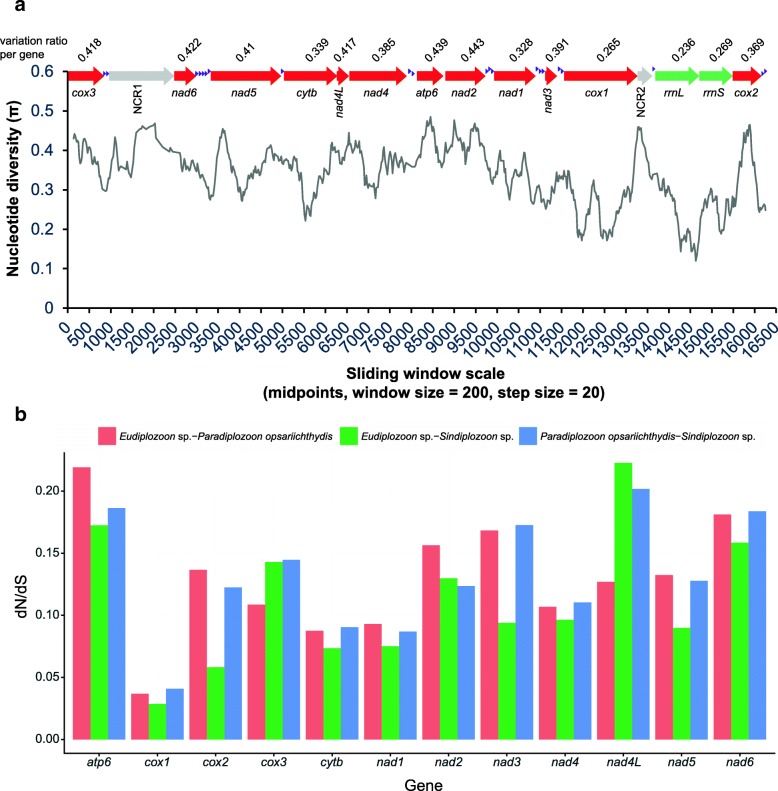
Selection pressures and sliding window analyses of the three diplozoid mitogenomes. **a** Sliding window analysis of the alignment of complete mtDNAs of the three diplozoids. *trnG* was removed from the analysis due to its position change. The black line represents the value of nucleotide diversity in a sliding window analysis (window size = 200 bp, step size = 20 bp, with the value inserted at its mid-point). Gene boundaries are indicated with a variation ratio per gene (above each gene). **b** Ratios of non-synonymous (dN) to synonymous (dS) substitution rates calculated from individual protein-coding genes of *Paradiplozoon opsariichthydis*, *Sindiplozoon* sp. and *Eudiplozoon* sp.

The sliding window analysis was conducted using the alignment of entire mitogenomes of the three studied diplozoids (*trnG* was removed from this analysis due to its position rearrangement). The plot of sequence variation ratio exhibited highly variable nucleotide diversity among the three aligned mitogenomic sequences, with Pi values for the 200 bp windows ranging from 0.12 to 0.485 (Fig. 5a). Genes with comparatively low sequence variability included *cox1* (0.265), *rrnL* (0.236), *rrnS* (0.269), *nad1* (0.328) and *cytb* (0.339), while genes with comparatively high sequence variability included *nad2* (0.443), *atp6* (0.439), *nad6* (0.422), *nad4L* (0.417) and *nad5* (0.41). These observations were also reflected in the results of dN/dS ratio (Fig. 5b) and average sequence identity (Table 1) analyses. However, similarity values strongly varied among different fragments of most genes (Fig. 5a).

### Phylogeny and gene order

**Fig. 6:**
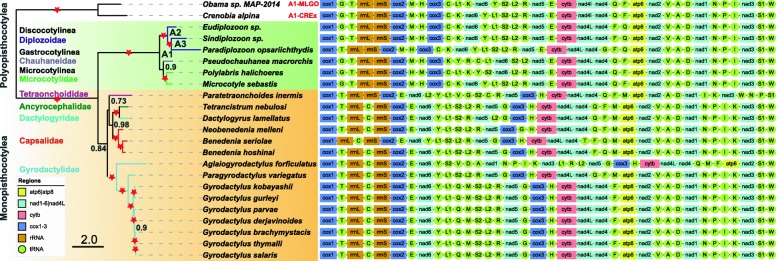
Phylogeny and mitogenomic architecture of the Monogenea class. The phylogram was constructed using CAT-MTART model based on concatenated amino acid sequences of 21 monogenean mitogenomes. *Crenobia alpina* and *Obama* sp. are outgroups. Scale bar corresponds to the estimated number of substitutions per site. Star symbol indicates that Bayesian posterior probability = 1.0, elsewhere values are shown above the nodes. A1, A2 and A3 are ancestral nodes. The top two gene orders are ancestral gene orders, as predicted for the A1 node by CREx and MLGO programs

Both ML analysis and BI analyses with MTART and CAT+MTART models produced phylograms with concordant branch topologies (Fig. 6). The Polyopisthocotylea clade was sub-divided into two clades, (Chauhaneidae+Microcotylidae) and Diplozoidae, both robustly supported (Bayesian posterior probabilities = 0.9 and 1, respectively, Fig. 6). As expected, the three diplozoids, *Eudiplozoon* sp., *P*. *opsariichthydis* and *Sindiplozoon* sp., constituted a monophyletic group with high support. The LG model, however, produced a different topology, with paraphyletic Diplozoidae (Additional file 6).

The gene orders of *Eudiplozoon* sp. and *Sindiplozoon* sp. were identical, while *P*. *opsariichthydis* exhibited a relocation of *trnG* to the position between *nad4* and *trnF*. Ancestral gene order reconstruction using both CREx and MLGO based on the optimal topology (the one generated by the MTART model) showed that the gene order as exhibited by *Eudiplozoon* sp. and *Sindiplozoon* sp. represents the ancestral state for the Diplozoidae family (i.e., the gene orders of A2 and A3 nodes are identical to these two species, Fig. 6). However, albeit the difference was minor (the position of *trnL1*), the two algorithms (CREx and MLGO) produced two different ancestral gene orders for the subclass Polyopisthocotylea. MLGO proposed the -*trnY*-*trnL1*-*trnS2*- order and CREx proposed the -*trnC*-*trnL1*-*trnK*- order (Fig. 6).

## Discussion

### Specimen identity

Identification of all three specimens was somewhat problematic, which additionally emphasizes the pressing need for a larger number of mitogenomes to be available in public databases. To support our morphological identification, we have provided high-resolution microscopic photographs of taxonomically important morphological traits (central hooks and clamps) for all three diplozoids (Additional files 7, 8 and 9). Both morphology (Additional file 7) and molecular data consistently identified the parasite collected from the hooksnout carp (*Opsariichthys bidens*) as a *Paradiplozoon* species. As the species in this genus are traditionally identified by their host [34], we identified it as *P. opsariichthydis*. However, as shown before [9], and visible in our ITS-2 rDNA analysis (Additional file 10), the status of these six Chinese *Paradiplozoon* species is highly doubtful. ITS-2 data strongly indicate that there is no evidence for the existence of host-specific species: the identity between our sequence and *P. opsariichthydis*, *P. parabramisi* Ling, 1973 and *P. hemiculteri* Ling, 1973 orthologs is 100% (GenBank numbers are shown in the Additional file 10).

*Eudiplozoon* sp. was morphologically similar to *E. nipponicum* (Additional file 8), the only recognized species in the genus, and it clustered with the six available *E. nipponicum* ITS-2 rDNA sequences (Additional file 10). Although this, as well as its host species and the locality where it was collected, indicates that it is indeed an *E. nipponicum* specimen, the ITS-2 rDNA sequence identities between our specimen and the remaining six *E. nipponicum* sequences were relatively low: 94% to 95%. This was also reflected in the topology of the phylogram, where our sequence was basal to the otherwise homogenous *E. nipponicum* clade (Additional file 10). In addition, the comparison with the only available *E. nipponicum cox1* sequence (AY009163) indicated an even lower identity value of 83%. Therefore, we suspect a possibility of the existence of a cryptic *Eudiplozoon* species. Further investigations of the *Eudiplozoon* population in Tangxun Lake are needed to establish whether the unusually low identities of ITS-2 and *cox1* sequences are a reflection of the existence of a cryptic species, as intrahost or sympatric speciation is more likely in monogeneans than in other parasitic groups [35–37], or merely an unusually high mutational rate of genomes (both nuclear and mitochondrial) of some sub-populations.

Host-specificity of the *Sindiplozoon* sp. specimens was very low; we found them in *Parabramis pekinensis*, *Spinibarbus hollandi* and black carp, so host information is not particularly relevant for the identification of this species. Morphological analysis (Additional file 9) suggests the highest similarity to *S. ctenopharyngodonis* among the six valid *Sindiplozoon* species [34]. However, the ITS-2 rDNA sequence identity between the studied species and *S. ctenopharyngodonis* (the only available sequence for the entire genus in the GenBank) was 97%. As five of the six valid *Sindiplozoon* species are non-represented in the GenBank, we can merely assign the specimen with certainty to the *Sindiplozoon* clade, but not to a particular species.

### Mitogenome architecture

We have successfully amplified the long non-coding region (LNR) of the mitogenome of *P. opsariichthydis*, so we know that the approximate size is 2000 bp, but we managed to sequence only 1429 bp. Considering that the non-sequenced gap was located near the TR region, we suspect that the sequencing of this region was likely to be hampered by such long repeat sequences; possibly by the secondary structures they form [38, 39]. The sequenced part of the LNR of *P. opsariichthydis* was comprised of only two TRs, but the sizes of both repeat units (244 bp repeat 1, 197 bp repeat 2) were the largest among the TRs reported in monogeneans so far [40–42]. A microsatellite-like TR of TA sequence (TA_*_) was also found in *Benedenia seriolae* (Yamaguti, 1934) [29]. Although the control region is generally considered to be difficult to locate in neodermatans (parasitic flatworms) [29], the presence of repeat regions is often associated with control regions [40, 43]. As they are present in all three diplozoids, we hypothesise that the described TA_*_ repeat regions are most likely embedded within the control regions. The fact that two mononucleotide adenine repeats, A_29_ and A_26_, were also detected in LNRs of *Eudiplozoon* sp. and *Sindiplozoon* sp., respectively, adds support to this hypothesis as they are also considered to be linked to the control region in fish [44].

Strong strand-specific nucleotide biases were also found in vertebrate [45] and pterobranch [46] mitogenomes. They are believed to be a consequence of strand-displacement mechanisms during the replication of mitogenomes [47]. It seems that strand-displacement modes were more prominent in cestodes and trematodes within the phylum Platyhelminthes, as they all exhibited comparatively high GC- and AT-skews (Fig. 1a-d). The three diplozoids appear to be outliers, with highest GC- and AT-skews, among the available monogenean mitogenomes (Fig. 1a-d). This finding suggests that slight GC- and AT-skews are not a synapomorphy for the class Monogenea.

Unorthodox start codons were reported in other neodermatans as well, such as ATT in *B. hoshinai* (Ogawa, 1984) and *Aglaiogyrodactylus forficulatus* (Kritsky, Vianna & Boeger 2007) [48, 49], TTG in *Paragyrodactylus variegatus* (You, King, Ye & Cone, 2014) [50] and GTT in *Hymenolepis diminuta* (Rudolphi, 1819) [51] (also see Additional file 11). The unusual codon frequency and the preference for G and/or T in the first site of anti-codons are a reflection of the selection for an efficient tRNA system [46, 52]. This further confirms the wobble versatility hypothesis (G pairs with T and C, T pairs with G and A in wobble pairing) [52], which suggests that GNN is the anticodon for NNY codon, and UNN is the anticodon for NNR and NNN codons. On this basis, the anticodon sequences with highest versatility (starting with G or T) were preferred in comparison to the anticodon sequences corresponding to the most frequent codons. In agreement with previous reports for the Nemertea phylum [53] and insects [54], our results indicate that there is no link between the abundance of codon families (Phe, Leu2 and Val are the most abundant) and the level of tRNA conservation (*trnP* is the most conserved) in diplozoid flatworms. Similar to gyrodactylids [55], nemerteans [53], and insects [54], some tRNAs have mismatched pairs in stems; e.g., T-T in the anticodon and acceptor stem of *trnQ*, A-G in the acceptor stem of *trnK*, and G-G in the anticodon stem of *trnF* (Figs. 3 and 4). They might be modified by the RNA editing process [56], or simply represent unusual pairings [57]. The high level of conservation in the DHU stem can be explained by the fact that DHU stem probably acts as a recognition site for the aminoacyl-tRNA synthetase [58]. Notably, the anticodon sequences of 21 tRNAs perfectly conformed to that of other monogeneans [15, 42, 50, 55, 59–61]. The only exception was *trnR*, which uses the ACG anticodon in *Eudiplozoon* sp. and *Sindiplozoon* sp., but TCG in *P. opsariichthydis* (Figs. 3 and 4). Although the shift of anticodon from TCG to ACG was also reported in *B. hoshinai* [59], this is most probably a homoplasy.

Sliding window analysis can identify regions with high nucleotide divergence, which is crucial for designing species-specific markers. These can be particularly useful in taxa where morphological identification is difficult and ambiguous, such as diplozoids. This information (variable similarity values among different fragments) can be used both for primer design and for functional studies. The *cox1* gene is often used as an universal barcode for species identification in general [62], as well as in monogeneans [63–66]. Therefore, our results, which show that *cox1* is the slowest evolving and least variable gene, indicate that its utility as a barcode for the family Diplozoidae, or even the entire class Monogenea, needs to be carefully tested and revised. If its resolution power indeed proves to be too low, we propose that fast-evolving *nad2*, *atp6* or *nad6* might be more suitable markers for future diagnostics/detection and population genetics studies of the family Diplozoidae.

Gene orders of the mitogenomes of *Eudiplozoon* sp., *P*. *opsariichthydis* and *Sindiplozoon* sp. conformed to the ground pattern of the subclass Polyopisthocotylea (pattern 3 in our recent paper [15]). Based on the ancestral gene order reconstruction results, the gene order of *P*. *opsariichthydis* was derived via a *trnG* transposition from the 3′ end of *cox1* to the 3′ end of *nad4*, and may be autapomorphic to the genus *Paradiplozoon*. Additionally, a position exchange between *trnF* and *trnQ* appears to have occurred in the common ancestor of the Diplozoidae family, distinguishing it from the ancestral gene order of the subclass Polyopisthocotylea. We hypothesise that the ancestral gene order (-*trnY*-*trnL1*-*trnS2*-) predicted by MLGO is more likely to be the ancestral state, as it appears to be more common in the available monogenean mitogenomes.

### Phylogeny

BI-LG and ML analyses produced incongruent topologies, with Diplozoidae resolved as paraphyletic in the former (Additional file 6). However, the paraphyly is likely to have been caused by the use of a sub-optimal LG + I + G + F model, as the optimal MTART model is not available in MrBayes. Regardless, this is rather intriguing (and therefore discussed here), because in our previous study BI and ML analyses of an almost identical dataset (minus the three new diplozoids) and using evolutionary identical models (BI – LG, ML – MTART) produced identical topologies [15]. Given that the two topologies are incongruent only regarding the three diplozoids, a possible explanation for this phenomenon would be comparatively long branches of all three diplozoids, but especially *P. opsariichthydis* (Additional file 6). We presume this is a reflection of the unusual nucleotide composition and amino acid usage of these three mitogenomes. A similar artefact, high GT content and a disproportionately long branch, was also reported in the unrelated *Rhabdopleura compacta* (Hincks, 1880) (Hemichordata) [46]. To attempt to resolve this problem by using a better-suited evolutionary model, we employed Phylobayes to conduct BI analysis with the optimal model (MTART) and a site-heterogeneous model with empirical matrixes CAT+MTART. Topologies produced by these two analyses are identical to the one produced by the ML analysis, which corroborates that incongruence was produced by the use of an unsuitable model LG. As this strategy, replacing the optimal MTART model with the closest model available in MrBayes, has been used in several studies [67–70], our results provide a note of caution regarding the interpretation of these results. We would encourage researchers to use PhyloBayes in such circumstances.

As the Diplozoidae + (Chauhaneidae + Microcotylidae) families belong to three different suborders, this also indicates that the suborder relationships are Discocotylinea + (Microcotylinea + Gastrocotylinea). This is incongruent with the polytomic topology inferred from 66 homologous series of morphological traits [32] and close relationship between Gastrocotylinea and Discocotylinea based on morphological characters and close relationship between Microcotylinea and Discocotylinea using large nuclear subunit ribosomal RNA gene [33]. The topology of the three Diplozoidae family genera, (*Eudiplozoon*, (*Sindiplozoon*, *Paradiplozoon*)), was also in disagreement with most previous studies [9, 12, 14], except for the ML topology in Gao et al. [9]. In our phylogram, *Paradiplozoon* (Chinese) formed a sister group with *Sindiplozoon* (with a robust support value). This close relationship is also reflected in the similarity of skewness for the full genome and its elements (PCGs) (Fig. 1a, b), as well as the similarity of codon and amino acid usage patterns (Fig. 1e, f). However, pairwise similarity (Table 1), secondary structures (Figs. 3 and 4) and similarity of skewness (Fig. 1c) for the 22 tRNAs, as well as similarity of skewness for the two rRNAs (Fig. 1d), did not reflect this close intergeneric relationship. This is in agreement with our observation that all RNA sequences of these mitogenomes exhibit slighter mutational biases than the PCGs (Fig. 1g), which may be associated with the mutational constraints imposed by the need for formation of stem-loop secondary structures (i.e., base pairing between A and T, G and C) [40].

## Conclusions

Unlike other monogeneans, mitogenomes of the three diplozoids exhibit enigmatic strand-specific nucleotide biases for the full genome, PCGs, tRNAs and rRNAs (Fig. 1a-d), as well as unusual codon and amino acid usage patterns for PCGs (Fig. 1e, f). In that aspect, they are more similar to cestodes and trematodes than to other monogenean flatworms. We suspect that this unusual bias has also caused disproportionately long branches of the three diplozoids in the phylogram. Intergeneric relationships within the family Diplozoidae produced in this study, as well as the interrelationships of Discocotylinea, Microcotylinea, and Gastrocotylinea suborders, are in disagreement with previous results based on small molecular markers and morphological traits [9, 12, 32, 33]. Therefore, our study might provide important new insights into the evolutionary history of the three genera and three suborders. However, as the three diplozoids exhibit unusual mitogenomic compositions, and as we had a limited number of diplozoid species at disposal for this analysis, we cannot reject with confidence the hypotheses put forward by the previous studies. Combined results of the sliding window, dN/dS ratio and average sequence identity analyses indicate that *nad2*, *atp6* and *nad6* are the most variable PCGs, whereas *cox1*, *nad1* and *cytb* are the most conserved PCGs. As *cox1* is often used as an universal barcode for species identification in general [62], as well as in monogeneans [63–66], our results suggest that its suitability for this task needs to be closely reassessed. Alternatively, we propose that *nad2*, *atp6* and *nad6* might be suitable markers for future diagnostics/detection and population genetics studies of the family Diplozoidae.

## Methods

### Specimen collection and identification

Multiple specimens belonging to three parasitic diplozoid species were obtained from gills of different fish species on three different locations in central China. The first species, *Paradiplozoon opsariichthydis*, was obtained from the Chinese hooksnout carp (*Opsariichthys bidens* Günther 1873) bought at a local market in Danjiangkou, Hubei Province (32°82′50″ – 33°81′50” N; 11°08′70″ – 11°18′60″ E) on 18/Apr/2015. The second species, *Eudiplozoon* sp., was obtained from a fish specimen belonging to the *Carassius auratus* complex [71, 72] Linnaeus, 1758 (henceforth referred to as gibel carp) caught by fishermen in Tangxun Lake, Hubei Province (30°22′56″ – 30°27′04” N; 114°20′14″ – 114°23′08″ E) on 22/May/2015. Samples belonging to the third species, *Sindiplozoon* sp., were collected from three different fish species, black carp (*Mylopharyngodon piceus* Richardson, 1846), *Parabramis pekinensis* (Basilewsky 1855), and *Spinibarbus hollandi* (Oshima 1919), caught by fishermen in the Mangtang Stream, Hunan Province (27°29′26″ – 27°29′31” N; 109°38′36″ – 109°38′51″ E) on 9/Jul/2016. As the host fishes were dead when we obtained from markets/fishermen, and parasitic invertebrates are not covered by animal welfare laws, thus no permission was needed. Parasites were identified morphologically under a light microscope according to the traits commonly used for the Diplozoidae family (central hooks and clamps) [3, 34]. As described in Civanova et al. [12], one of the two opisthaptors of each parasite was cut off and soaked in 10% sodium dodecyl sulfate for 60 min. After washing in distilled water, the opisthaptor was mounted on a microscope slide and fixed with a mixture of ammonium picrate and glycerin. Additionally, to confirm the taxonomic identity using molecular data, DNA was extracted from one of the two anterior parts of the parasite body, and used to amplify *ITS-2 rDNA* gene using the universal eukaryotic primers [73]: D (5′-GGCTYRYGGNGTCGATGAAGAACGCAG-3′) and B1 (5′-GCCGGATCCGAATCCTGGTTAGTTTCTTTTCCT-3′). Finally, the remaining incomplete parasite bodies (one anterior part and one opisthaptor) of the three species were permanently stored as vouchers in 100% ethanol at the Parasitology and Coevolution Lab (room number 511), Institute of Hydrobiology, Chinese Academy of Sciences, Wuhan, China (Accession No.: IHB20180620001 for *P. opsariichthydis*, IHB20180620002 for *Eudiplozoon* sp., and IHB20180620003 for *Sindiplozoon* sp.).

### DNA extraction, amplification and sequencing

Degenerate primer pairs (Additional file 12) were designed to match the generally conserved regions of mitochondrial genes and used to amplify and sequence these short conserved fragments. Based on these fragments, we then designed specific primers for the amplification and sequencing of the remaining mitogenomic sequences in several PCR steps. PCR was performed in a 20 μl reaction mixture, containing 7.4 μl dd H_2_O, 10 μl 2 × PCR buffer (Mg^2+^, dNTP plus, Takara, Dalian, China), 0.6 μl of each primer, 0.4 μl r*Taq* polymerase (250 U, Takara), and 1 μl of DNA template. Amplification was conducted under the following conditions: initial denaturation at 98 °C for 2 min, followed by 40 cycles at 98 °C for 10 s, 48–60 °C for 15 s, 68 °C for 1 min/kb, and a final extension at 68 °C for 10 min. PCR products were sequenced bi-directionally on an ABI 3730 automatic sequencer using Sanger method. During the sequencing we paid close attention to electropherograms, carefully examining them for double peaks, or any other sign of the existence of two different sequences. All obtained fragments were BLASTed [74] to confirm that the amplicon is the actual target sequence.

### Sequence annotation and analyses

After quality-proofing of the obtained fragments, the three mitogenomic sequences were assembled manually in a stepwise manner with the help of DNAstar v7.1 program [75]. The sequence of the *P. opsariichthydis* mitogenome could not be assembled into a full circular genome due to a gap of around 2000 bp in the long non-coding region (LNR). All mitogenomes were annotated mainly following the procedure described before [15, 25, 30, 42]. To determine the approximate boundaries of genes, all three mitogenomic sequences were aligned against a selected reference mitogenome, *Pseudochauhanea macrorchis* (Liu, Zhang & Lin, 2001, NC_016950) [32], using MAFFT [76] integrated into Geneious [77]. Protein-coding genes (PCGs) were found by searching for ORFs (employing genetic code 9, echinoderm mitochondrial) and checking nucleotide alignments against the reference genome in Geneious. All 22 tRNAs were identified using ARWEN [78] and MITOS [79] web servers, and their secondary structures were visualized according to these results. Exceptions were *trnC* and *trnK* of *Eudiplozoon* sp., which were found by the alignment with orthologs in *P. opsariichthydis* and *Eudiplozoon* sp. mitogenomes. The two rRNAs, *rrnL* and *rrnS*, were also initially identified using MITOS, and their precise boundaries determined via a comparison with homologs in Geneious. A home-made GUI-based program, MitoTool [80], was used to create GenBank submission files and tables with statistics for mitogenomes by parsing the annotations recorded in Word documents. Nucleotide (nt) composition and amino acid (aa) composition tables of all available monogenean mitogenomes were generated by MitoTool, and then used to make the broken line graph of skewness and G + T content for nt, as well as stacked bar chart for amino acids in ggplot2 [81]. Codon usage and relative synonymous codon usage (RSCU) for twelve protein-encoding genes (PCGs) of the six polyopisthocotylids characterized so far were computed and sorted using MitoTool, and finally imported into ggplot2 to draw the RSCU figure. All scatter diagrams for the principal component analysis (PCA) and nucleotide skews were also drawn by ggplot2. Input files for the PCA of the codon usage pattern, as well as the aa usage pattern and the analyses of nucleotide skews for all available flatworm (free-living and parasitic) mitogenomes, were also generated by MitoTool. PASW 18.0 [82] was used to conduct principal component analysis and generate data for the scatter diagram. Non-synonymous (dN) / synonymous (dS) mutation rate ratios among the 12 PCGs of the three studied diplozoid mitogenomes were calculated with KaKs_Calculator [83] utilizing a modified Yang–Nielsen algorithm. DnaSP v5 [84] was employed to conduct the sliding window analysis: a sliding window of 200 bp and a step size of 20 bp was implemented to estimate the nucleotide divergence Pi between the mitogenomes of *Sindiplozoon* sp., *P. opsariichthydis* and *Eudiplozoon* sp.. Tandem Repeats Finder [85] was employed to find tandem repeats in the non-coding regions, and their secondary structures were predicted by Mfold software [86].

### Phylogenetic analyses

Phylogenetic analyses were conducted using the three newly sequenced diplozoid mitogenomes and all 18 monogenean mitogenomes available in the GenBank (9/Nov/2017). As suggested in our previous study [42], two species of the order Tricladida, *Crenobia alpina* (Dana, 1766) (KP208776) and *Obama* sp. MAP-2014 (NC_026978), were used as outgroups, thus adding up to 23 mitogenomes in total (Additional file 13). Fasta files with nucleotide sequences for twelve protein-coding genes (PCGs) were extracted from GenBank files and translated into amino acid sequences (employing genetic code 9, echinoderm mitochondrial) using MitoTool as described before [15, 25, 30, 42, 87]. Amino acid sequences were aligned in batches and ambiguously aligned fragments removed from the concatenated alignment using MAFFT [76] and Gblocks 0.91b [88] (respectively) integrated into another GUI-based program compiled by us, BioSuite [89]. Phylogenetic analyses were performed using amino acid sequences of 12 PCGs. Phylogenetic analyses were conducted using two different algorithms: maximum likelihood (ML) and Bayesian inference (BI). Based on the Akaike information criterion, MTART+I + G + F was chosen as the optimal evolutionary model for the phylogenetic analysis, using ProtTest [90]. ML analysis was conducted in RaxML [91] using a ML + rapid bootstrap algorithm with 1000 replicates. As described in our recent paper [15], BI analysis was firstly carried out in MrBayes 3.2.6 [92] with default settings, and 5 × 10^6^ metropolis-coupled MCMC generations, using a sub-optimal model LG + I + G + F (the closest implemented model to the optimal MTART+I + G + F). Stationarity was considered to be reached when the average standard deviation of split frequencies was below 0.01, ESS (estimated sample size) value was above 200, and PSRF (potential scale reduction factor) approached 1. Bayesian inference analyses with empirical model MTART and site-heterogeneous model with empirical matrixes CAT+MTART were conducted using PhyloBayes MPI 1.5a [93]. For each analysis, two MCMC chains were run after the removal of invariable sites from the alignment, and the analysis was stopped when the conditions considered to indicate a good run (PhyloBayes manual) were reached: maxdiff < 0.1 and minimum effective size > 300. Sequence alignment matrices and the resultant trees were deposited in TreeBASE repository [94] under the accession number 21941. Phylograms and gene orders were visualized and annotated by iTOL [95] with the help of several dataset files generated by MitoTool, as described in our recent papers [15, 30, 96]. Finally, based on the resultant optimal phylogram, we performed an ancestral character state inference for the gene orders in the subclass Polyopisthocotylea using MLGO web server [97] (ML algorithm) and TreeREx [98] (common interval algorithm).

## Additional files

Additional file 1: Visual representation of the circular mitochondrial genomes of *Paradiplozoon opsariichthydis*, *Sindiplozoon* sp. and *Eudiplozoon* sp.. Protein-coding genes (12) are red, tRNAs (22) are yellow, rRNAs (2) are green, and non-coding regions are grey. (PDF 247 kb)

Additional file 2: Nucleotide composition and skewness comparison of different elements of the mitochondrial genomes of *Paradiplozoon opsariichthydis*, *Sindiplozoon* sp. and *Eudiplozoon* sp.. NCR: non-coding region; PCG: protein-coding gene. (XLSX 16 kb)

Additional file 3: Relative synonymous codon usage (RSCU) of six polyopisthocotylid mitogenomes. Codon families are labelled on the x-axis. Values on the top of the bars refer to amino acid usage. (PDF 273 kb)

Additional file 4: Summary of multiple alignments of tRNA genes in diplozoid mitogenomes. ALN: alignment; INUC: percent of identical nucleotides. (XLSX 12 kb)

Additional file 5: Secondary structure of the small non-coding regions in the mitogenomes of *Paradiplozoon opsariichthydis*, *Sindiplozoon* sp. and *Eudiplozoon* sp., and tandem repeats in the large non-coding regions in the genomes of *Paradiplozoon opsariichthydis* and *Sindiplozoon* sp.. (PDF 616 kb)

Additional file 6: Phylogenetic trees based on all four analyses. (PDF 411 kb)

Additional file 7: Light micrographs of the central hook and clamps of *Paradiplozoon opsariichthydis*. **a.** A row of clamps and one central hook under a 20× light microscope. **b.** Central hooks and clamps under a 10× light microscope. **c.** Another row of clamps and central hook under a 20× light microscope. **d.** Central hook and part of clamps under a 40× light microscope. (TIF 15208 kb)

Additional file 8: Light micrographs of central hooks and clamps of *Eudiplozoon* sp.. **a.** Central hooks and clamps under a 20× light microscope. **b.** Central hooks and clamps under a 20× confocal laser scanning microscope. **c.** A row of clamps and one central hook under a 40× light microscope. **d.** Another row of clamps and central hook under a 40× light microscope. (TIF 13034 kb)

Additional file 9: Light micrographs of central hooks and clamps of *Sindiplozoon* sp.. **a.** A row of clamps under a 20× light microscope. **b.** Another row of clamps under a 20× light microscope. **c.** All clamps under a 20× confocal laser scanning microscope. **d.** Central hooks under a 40× light microscope. (TIF 15007 kb)

Additional file 10: Phylogenetic tree based on 69 ITS-2 rDNA sequences using maximum-likelihood algorithm. (PDF 313 kb)

Additional file 11: General statistics (length and codons) for mitochondrial protein-coding genes and rRNAs of 21 monogeneans. Abbreviations of species name are combined initials of the genus and species name. (XLSX 13 kb)

Additional file 12: Primers used to amplify and sequence the mitochondrial genomes of *Paradiplozoon opsariichthydis*, *Sindiplozoon* sp. and *Eudiplozoon* sp.. (XLSX 11 kb)

Additional file 13: The list of monogenean species and outgroups used for comparative mitogenomic and phylogenetic analyses. (XLSX 15 kb)

## Abbreviations

aa: amino acid
BI: Bayesian inference
cbc: compensatory base changes
dN: Non-synonymous mutation rate
dS: synonymous mutation rate
ITS: Internal transcribed spacer
LNR: Long non-coding region
ML: Maximum likelihood
nt: nucleotide
ORF: Open reading frame
PCA: Principal component analyses
PCGs: Protein-encoding genes
RSCU: Relative synonymous codon usage
SNR: Short non-coding region
TA_*_: Tandem repeat of the sequence TA
TR: Tandem repeat
