# Supplementary figures and images for: Three new Diplozoidae mitogenomes expose unusual compositional biases within the Monogenea class: implications for phylogenetic studies
Source: BMC Evol Biol. 2018 Sep 3;18:133. doi: 10.1186/s12862-018-1249-3 (PMC6122551; doi:10.1186/s12862-018-1249-3)

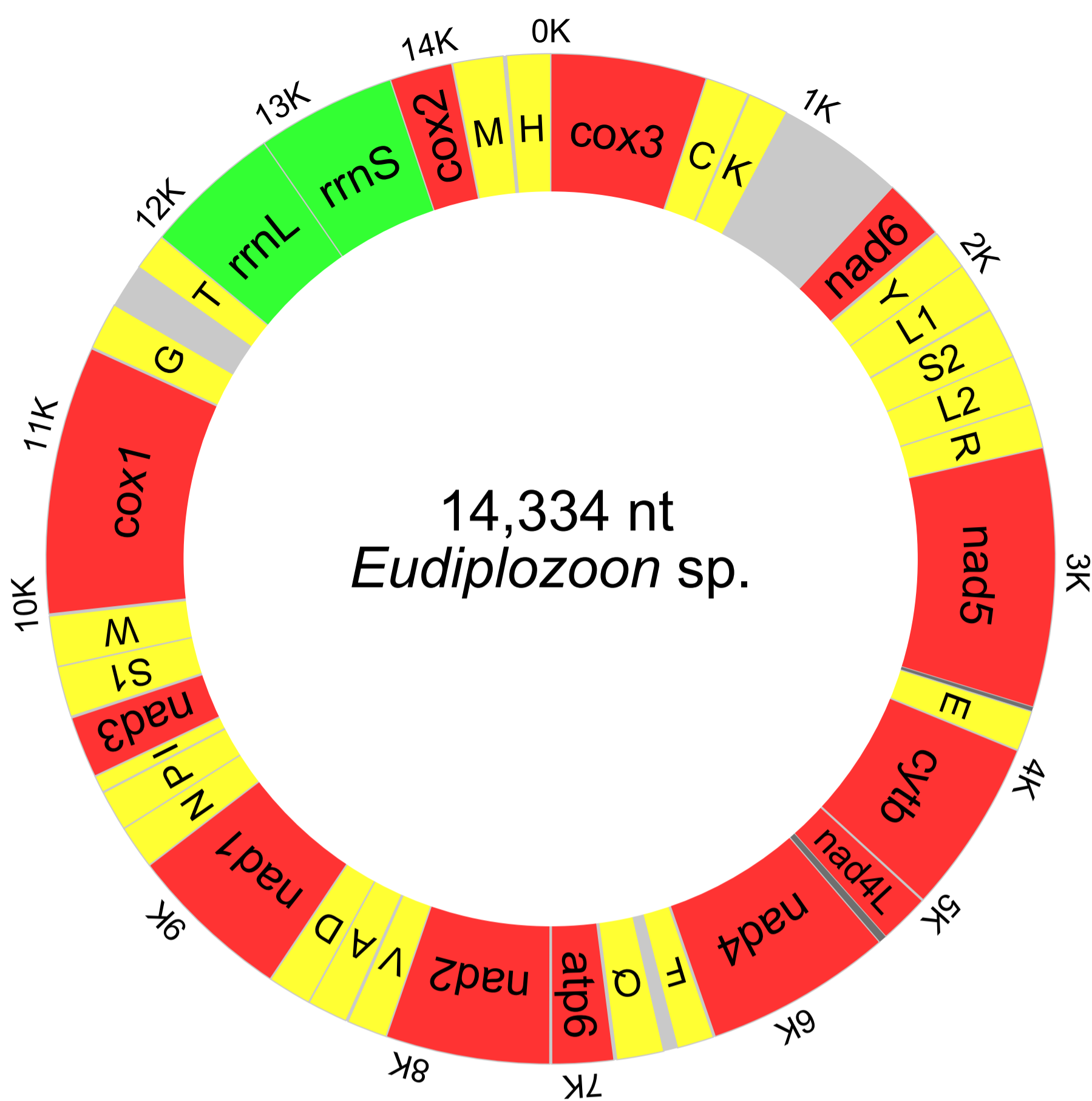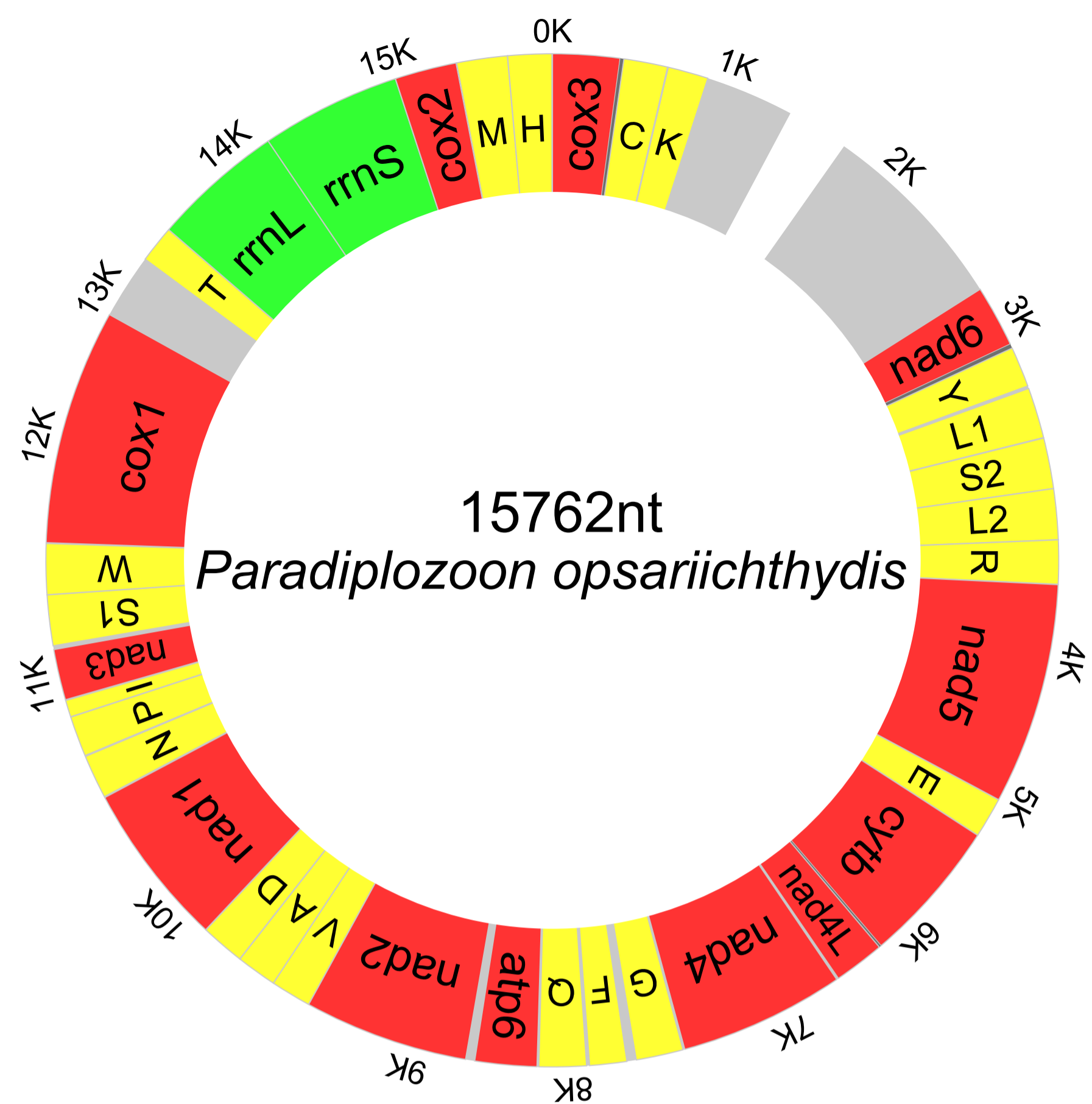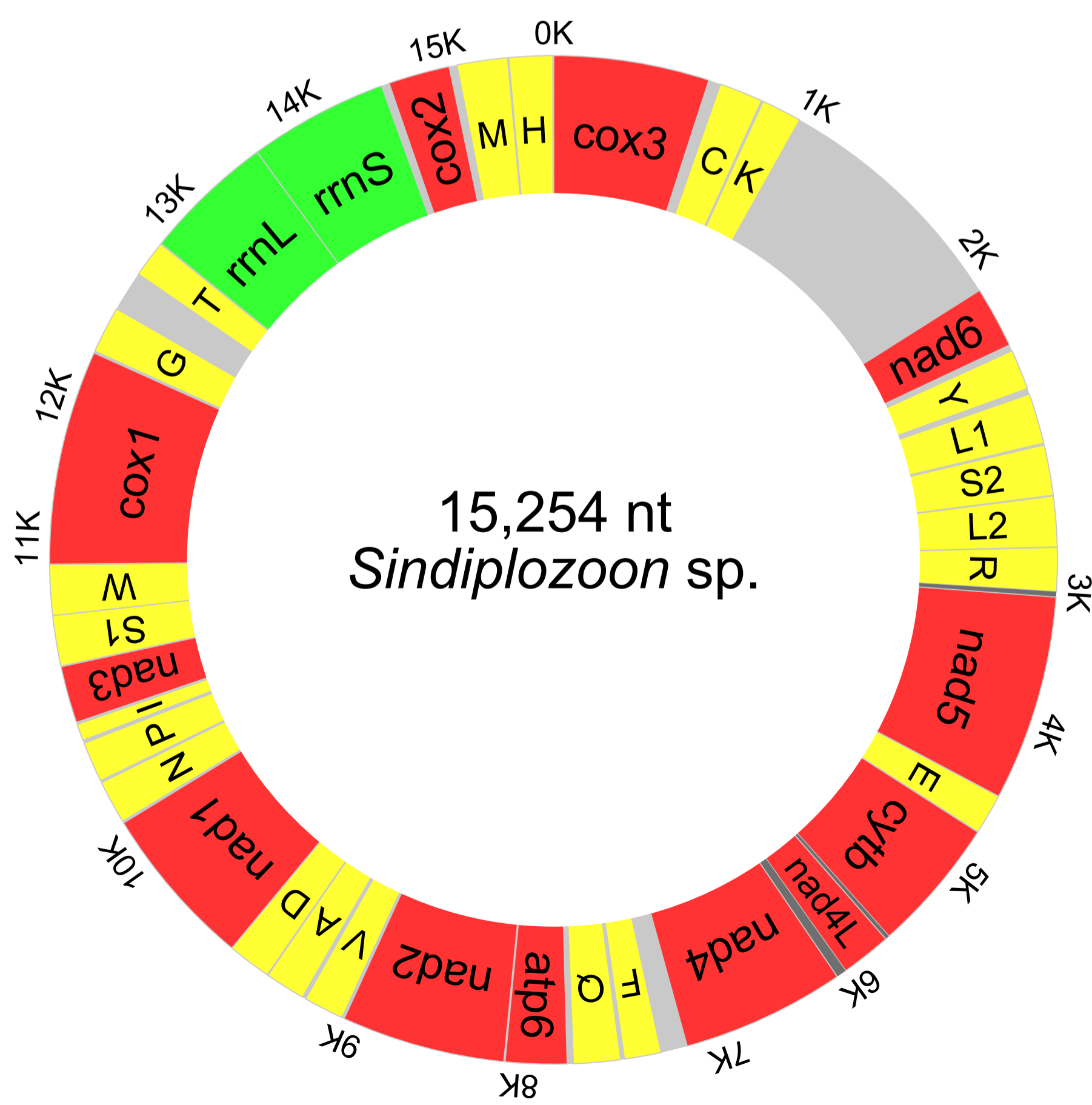

Supplement: Supplementary file 1 — Visual representation of the circular mitochondrial genomes of Paradiplozoon opsariichthydis, Sindiplozoon sp. and Eudiplozoon sp.. Protein-coding genes (12) are red, tRNAs (22) are yellow, rRNAs (2) are green, and non-coding regions are grey. (PDF 247 kb) [file 12862_2018_1249_MOESM1_ESM.pdf]

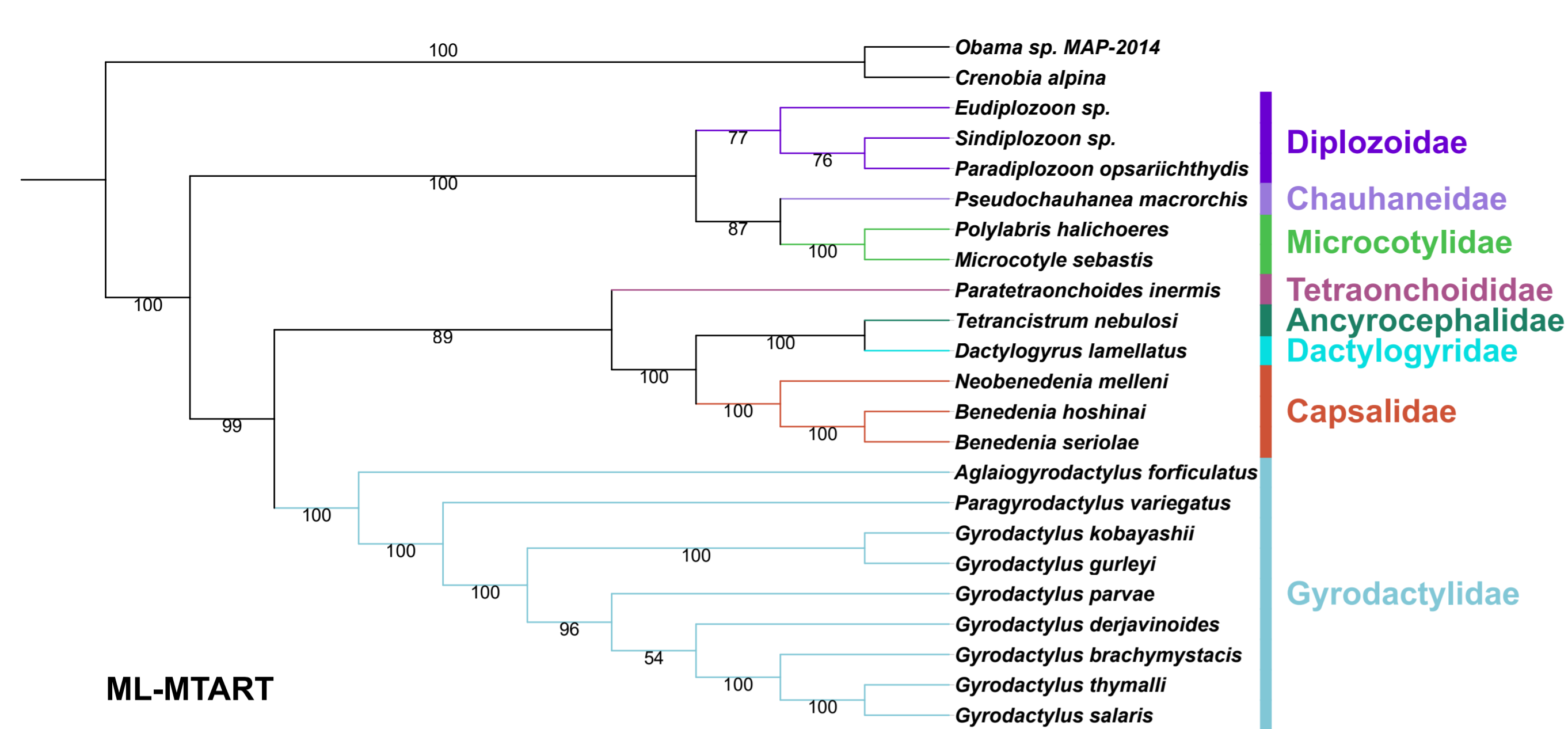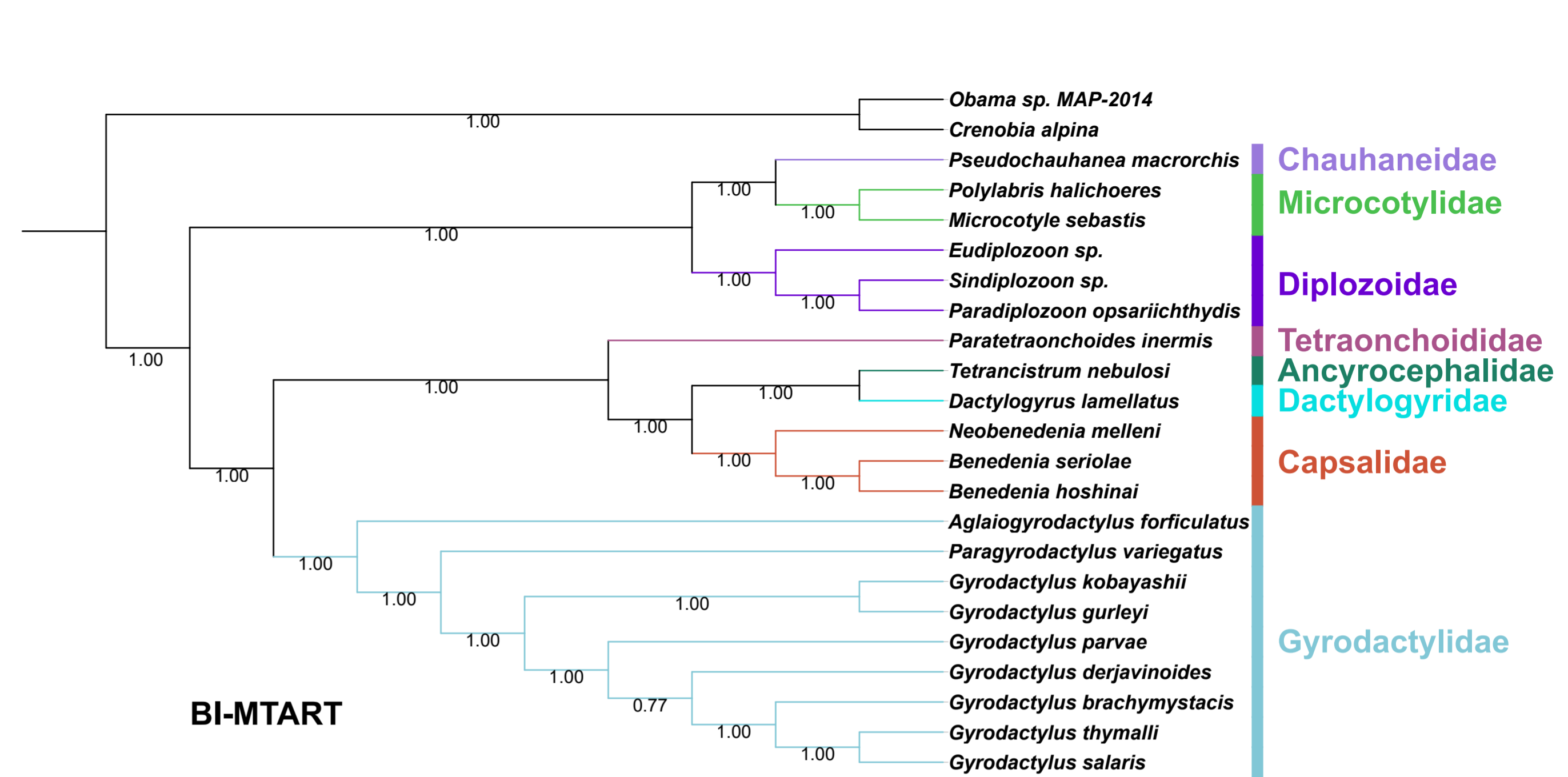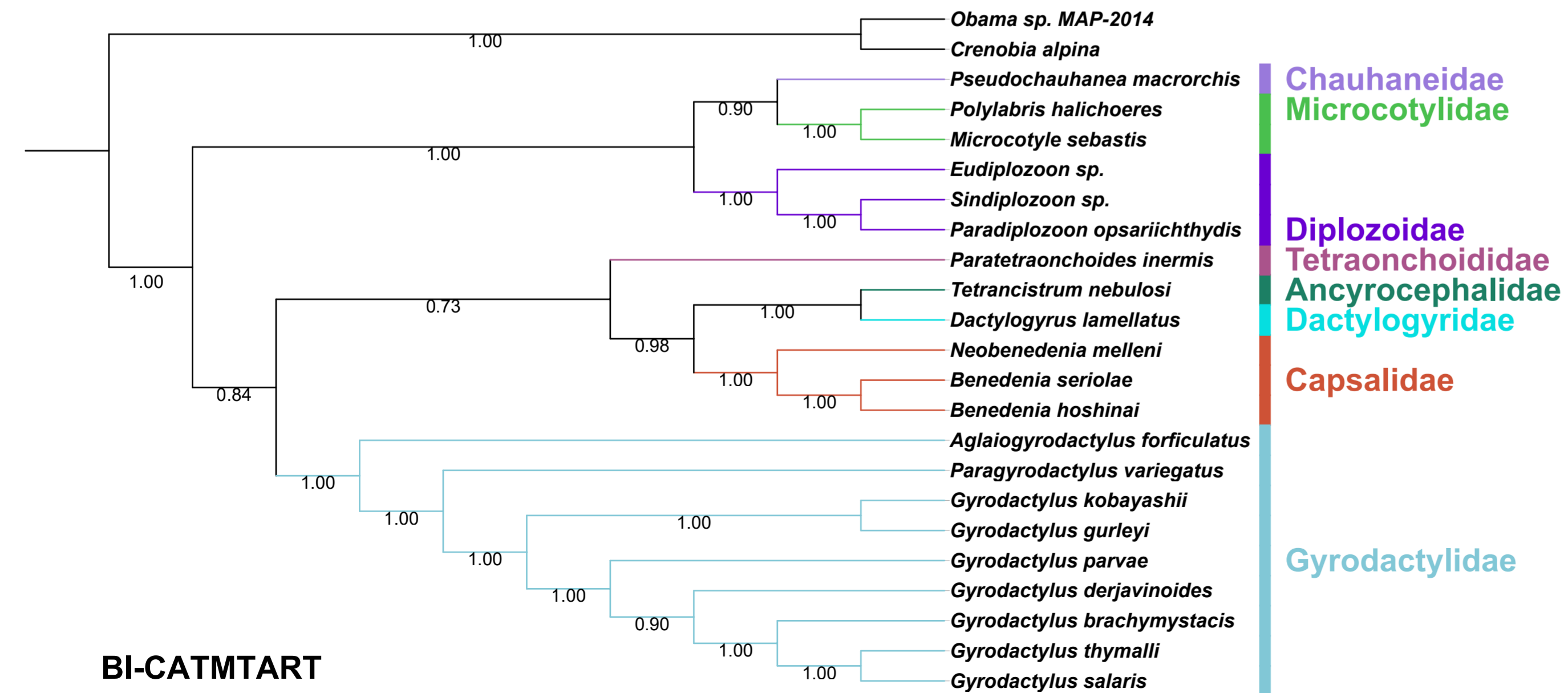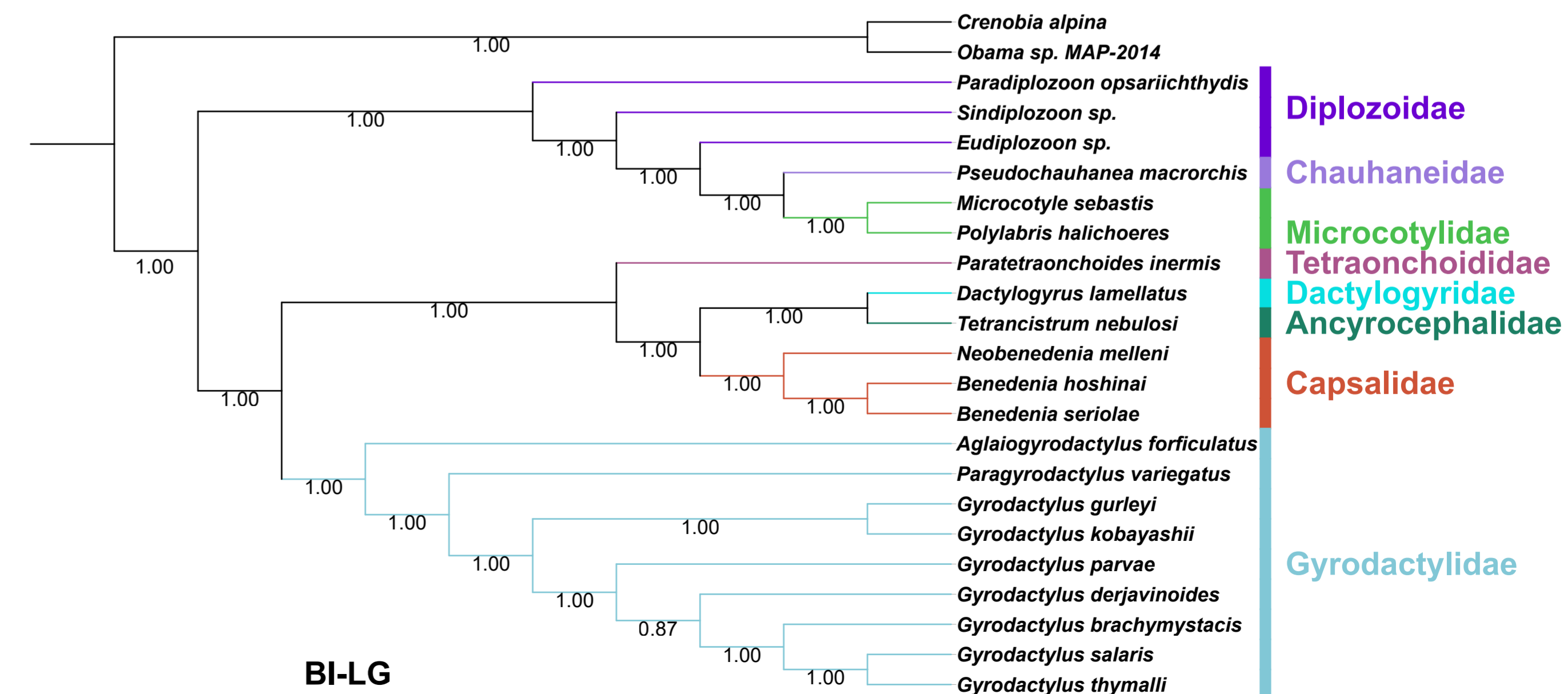

Supplement: Supplementary file 6 — Phylogenetic trees based on all four analyses. (PDF 411 kb) [file 12862_2018_1249_MOESM6_ESM.pdf]

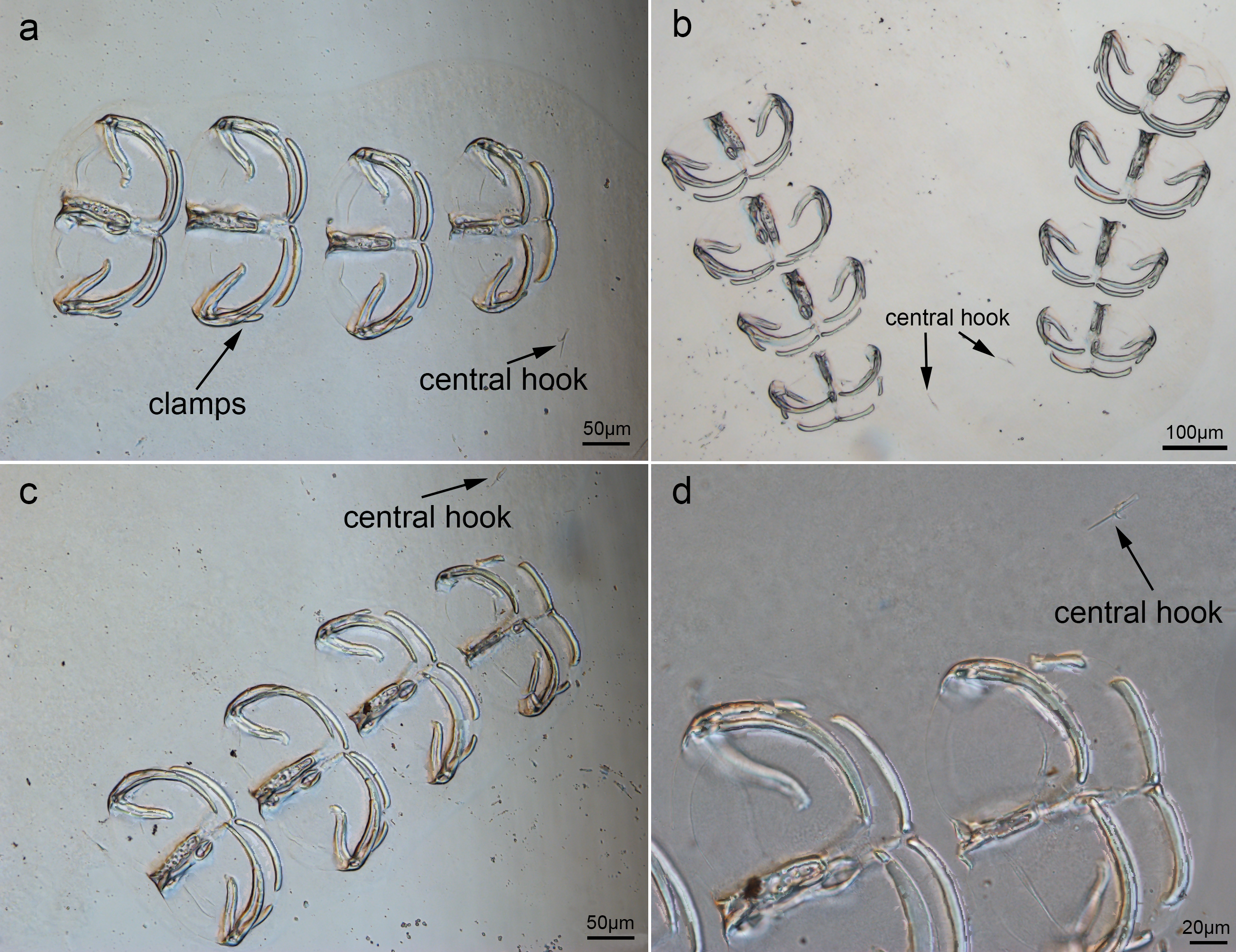

Supplement: Supplementary file 7 — Light micrographs of the central hook and clamps of Paradiplozoon opsariichthydis. a. A row of clamps and one central hook under a 20× light microscope. b. Central hooks and clamps under a 10× light microscope. c. Another row of clamps and central hook under a 20× light microscope. d. Central hook and part of clamps under a 40× light microscope. (TIF 15208 kb) [file 12862_2018_1249_MOESM7_ESM.tif]

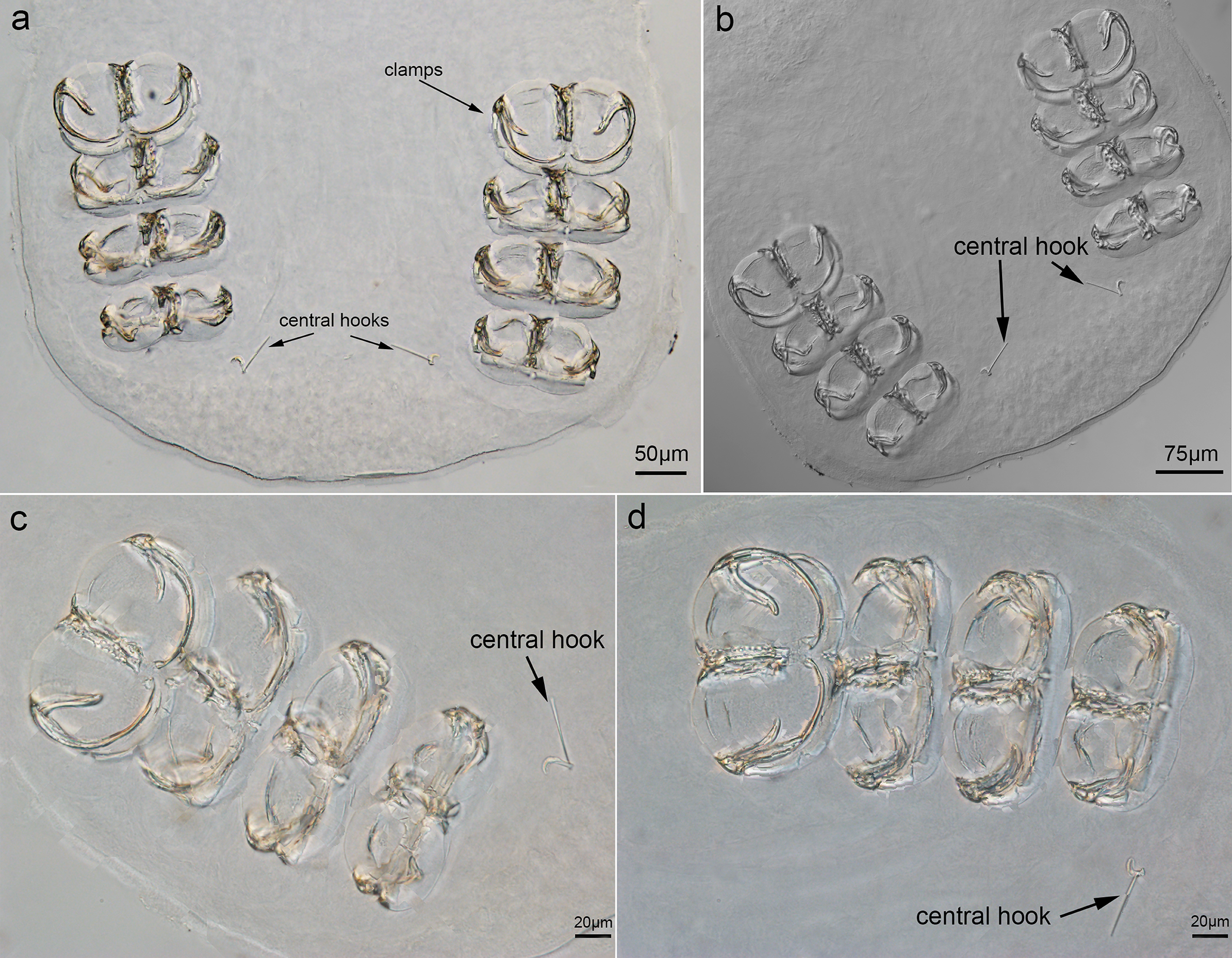

Supplement: Supplementary file 8 — Light micrographs of central hooks and clamps of Eudiplozoon sp.. a. Central hooks and clamps under a 20× light microscope. b. Central hooks and clamps under a 20× confocal laser scanning microscope. c. A row of clamps and one central hook under a 40× light microscope. d. Another row of clamps and central hook under a 40× light microscope. (TIF 13034 kb) [file 12862_2018_1249_MOESM8_ESM.tif]

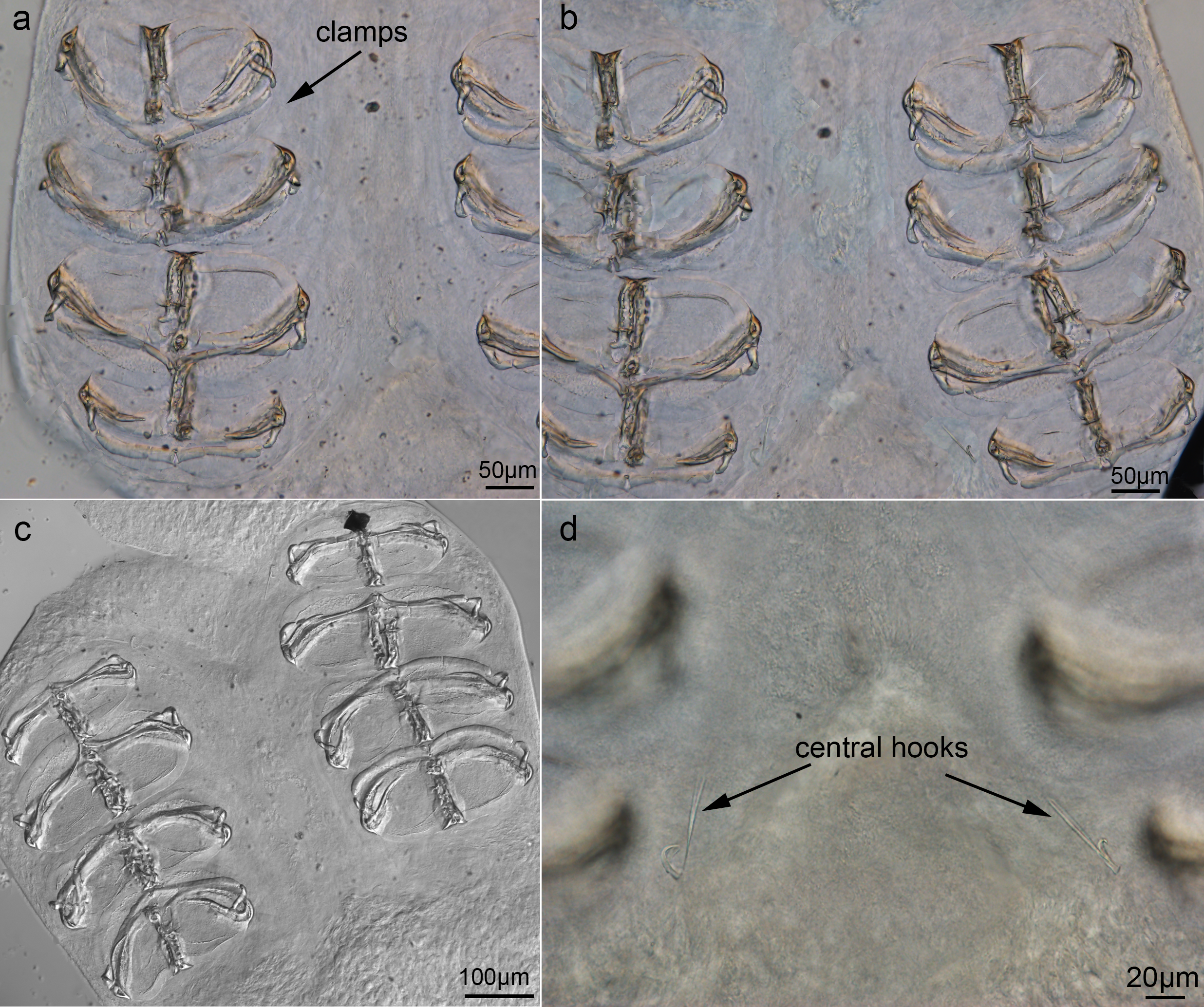

Supplement: Supplementary file 9 — Light micrographs of central hooks and clamps of Sindiplozoon sp.. a. A row of clamps under a 20× light microscope. b. Another row of clamps under a 20× light microscope. c. All clamps under a 20× confocal laser scanning microscope. d. Central hooks under a 40× light microscope. (TIF 15007 kb) [file 12862_2018_1249_MOESM9_ESM.tif]

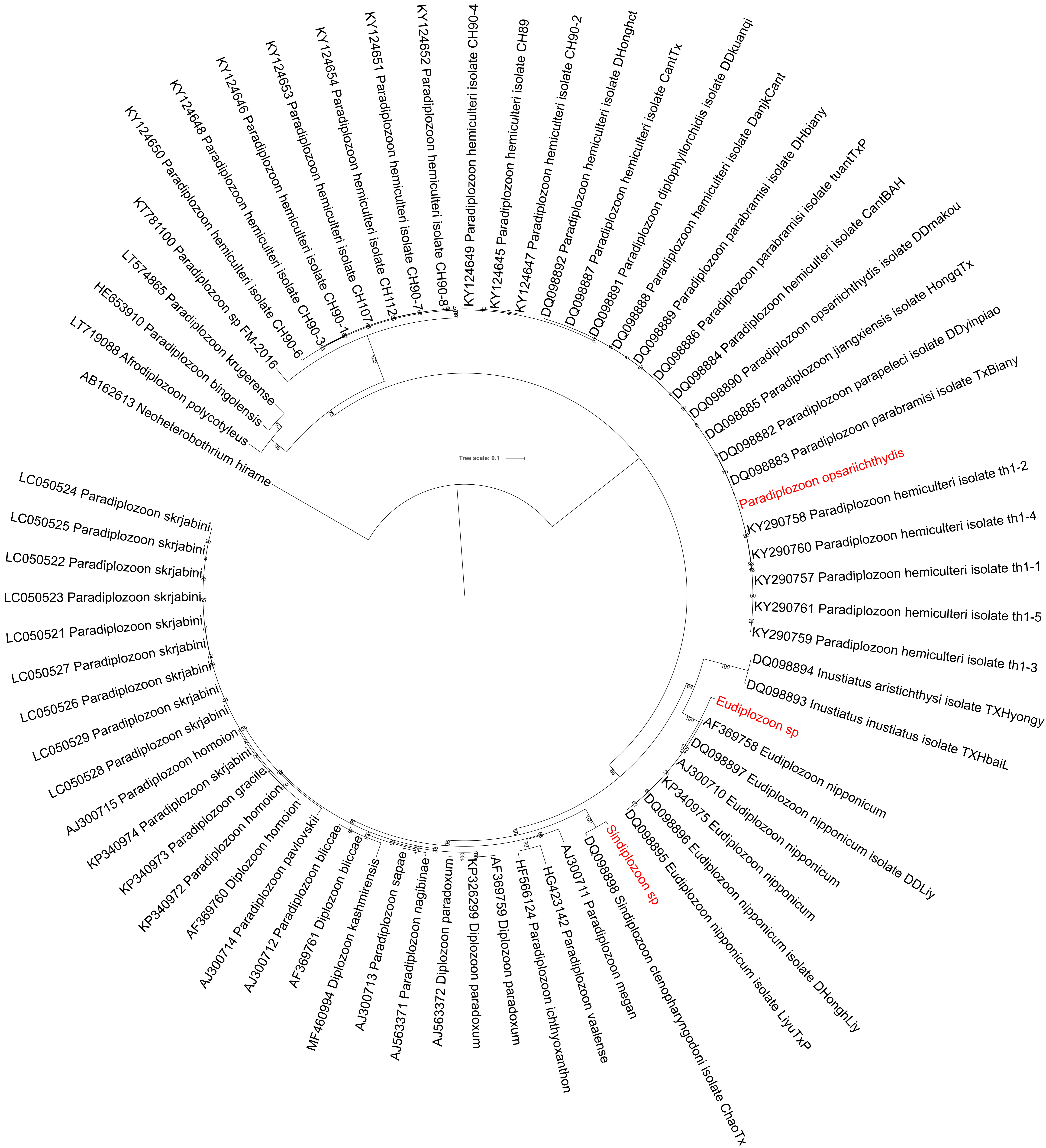

Supplement: Supplementary file 10 — Phylogenetic tree based on 69 ITS-2 rDNA sequences using maximum-likelihood algorithm. (PDF 313 kb) [file 12862_2018_1249_MOESM10_ESM.pdf]
