# Supplementary material for: Three new Diplozoidae mitogenomes expose unusual compositional biases within the Monogenea class: implications for phylogenetic studies
Source: BMC Evol Biol. 2018 Sep 3;18:133. doi: 10.1186/s12862-018-1249-3 (PMC6122551; doi:10.1186/s12862-018-1249-3)
Supplement: Supplementary file 3 — Relative synonymous codon usage (RSCU) of six polyopisthocotylid mitogenomes. Codon families are labelled on the x-axis. Values on the top of the bars refer to amino acid usage. (PDF 273 kb) [file 12862_2018_1249_MOESM3_ESM.pdf]

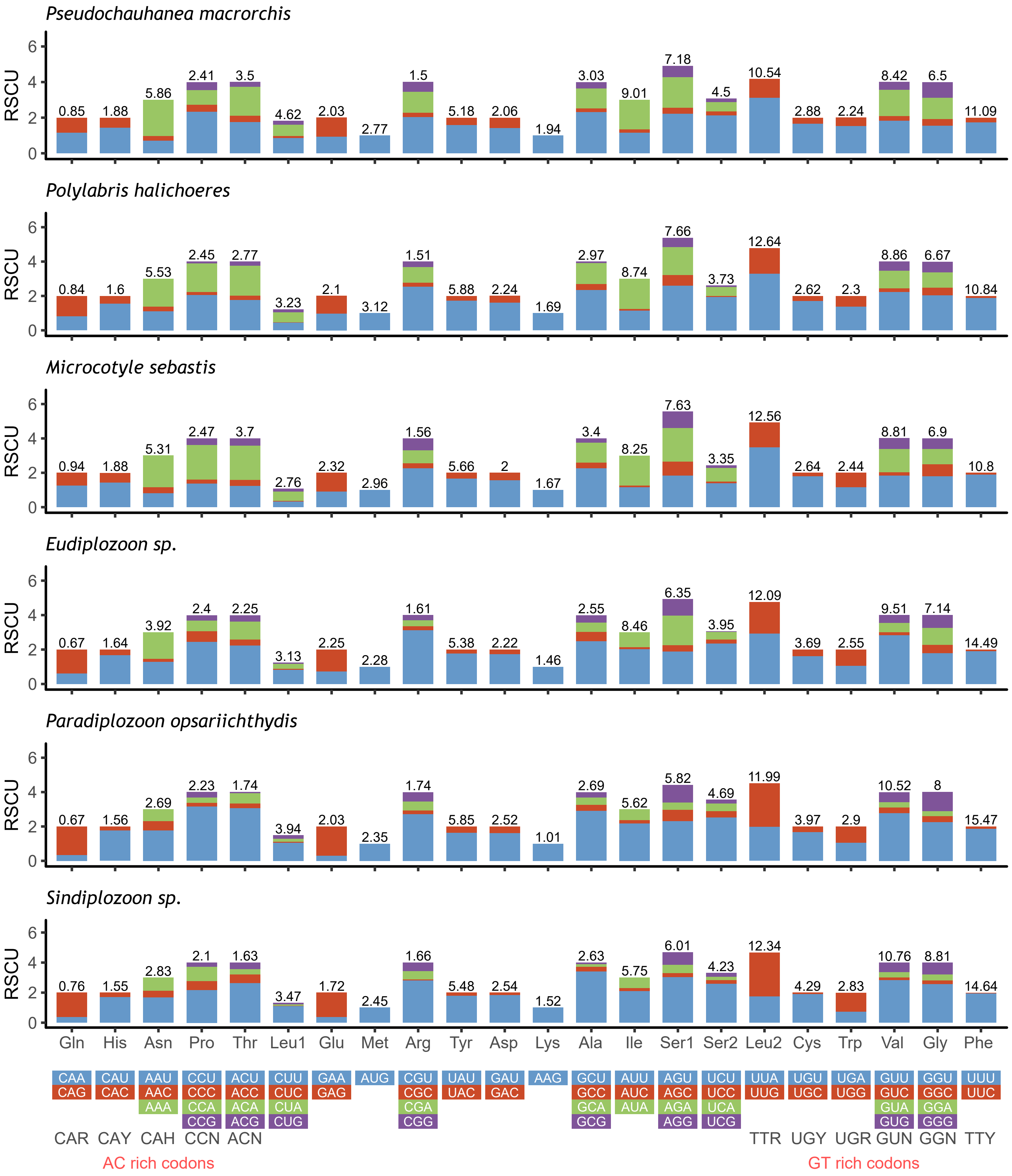

Pseudochauhanea macrorchis
Polylabris halichoeres
Microcotyle sebastis
Eudiplozoon sp.
Paradiplozoon opsariichthydis
Sindiplozoon sp.
RSCU
Gln His Asn Pro Thr Leu1 Glu Met Arg Tyr Asp Lys Ala Ile Ser1 Ser2 Leu2 Cys Trp Val Gly Phe
CAA CAG
CAU CAC
AAU AAC AAA
CCU CCC CCA CCG
ACU ACC ACA ACG
CUU CUC CUA CUG
GAA GAG
AUG
CGU CGC CGA CGG
UAU UAC
GAU GAC
AAG
GCU GCC GCA GCG
AUU AUC AUA
AGU AGC AGA AGG
UCU UCC UCA UCG
UUA UUG
UGU UGC
UGA UGG
GUU GUC GUA GUG
GGU GGC GGA GGG
UUU UUC
CAR CAY CAH CCN ACN
TTR UGY UGR GUN GGN TTY
AC rich codons
GT rich codons
