# Supplementary material for: Three new Diplozoidae mitogenomes expose unusual compositional biases within the Monogenea class: implications for phylogenetic studies
Source: BMC Evol Biol. 2018 Sep 3;18:133. doi: 10.1186/s12862-018-1249-3 (PMC6122551; doi:10.1186/s12862-018-1249-3)
Supplement: Supplementary file 5 — Secondary structure of the small non-coding regions in the mitogenomes of Paradiplozoon opsariichthydis, Sindiplozoon sp. and Eudiplozoon sp., and tandem repeats in the large non-coding regions in the genomes of Paradiplozoon opsariichthydis and Sindiplozoon sp.. (PDF 616 kb) [file 12862_2018_1249_MOESM5_ESM.pdf]

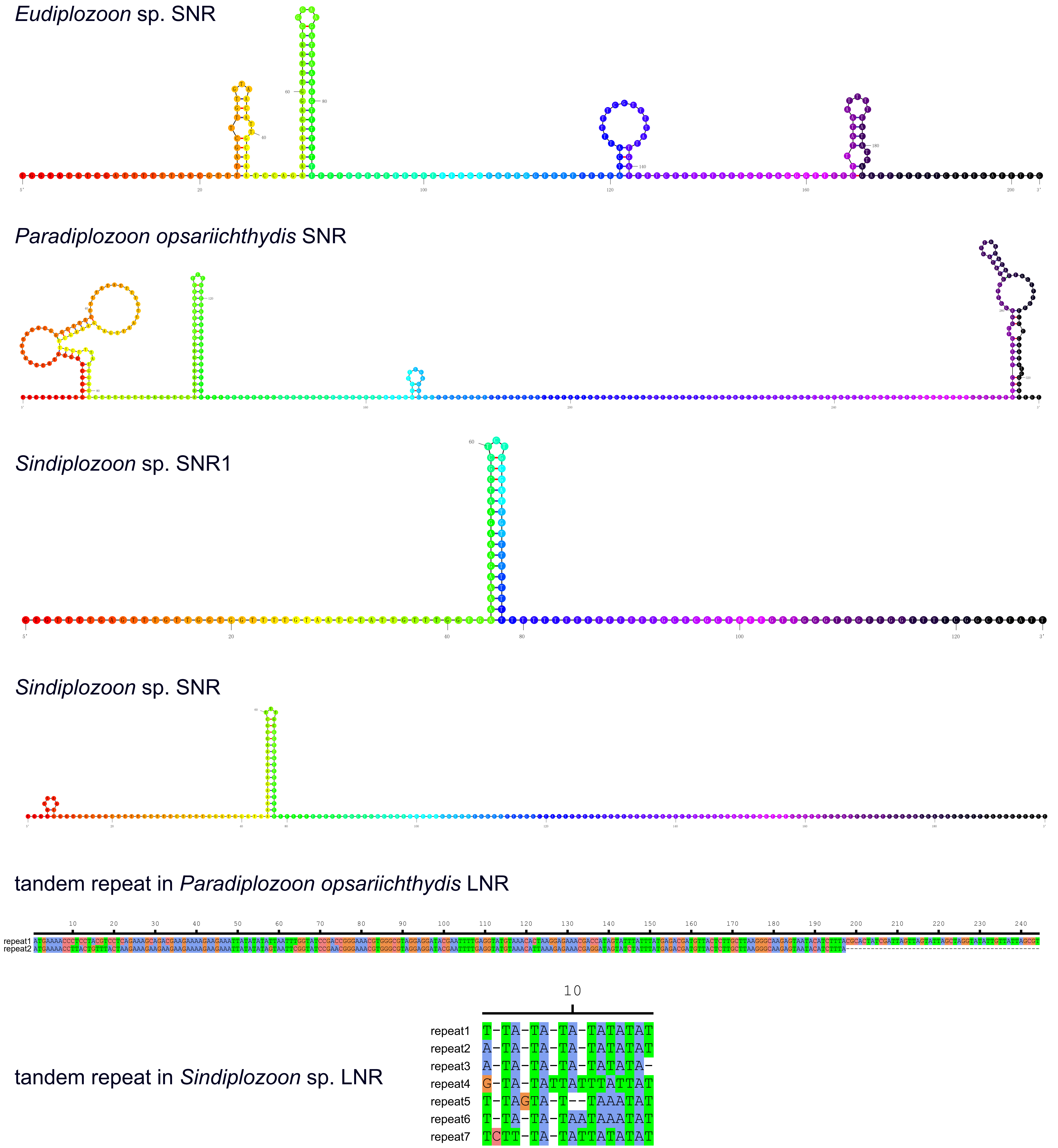

Eudiplozoon sp. SNR
Paradiplozoon opsariichthydis SNR
Sindiplozoon sp. SNR1
Sindiplozoon sp. SNR
tandem repeat in *Paradiplozoon opsariichthydis* LNR
repeat1
repeat2
tandem repeat in *Sindiplozoon* sp. LNR
10
repeat1 T-TA-TA-TA-TATATAT
repeat2 A-TA-TA-TA-TATATAT
repeat3 A-TA-TA-TA-TATATA-
repeat4 G-TA-TATTATTTATTAT
repeat5 T-TAGTA-T--TAAATAT
repeat6 T-TA-TA-TAATAAATAT
repeat7 TCTT-TA-TATTATATAT
